# Genomic Association vs. Serological Determination of ABO Blood Types in a Chinese Cohort, with Application in Mendelian Randomization

**DOI:** 10.3390/genes12070959

**Published:** 2021-06-24

**Authors:** Mengqiao Wang, Jiaqi Gao, Jin Liu, Xing Zhao, Yi Lei

**Affiliations:** 1 Department of Epidemiology and Biostatistics, West China School of Public Health and West China Fourth Hospital, Sichuan University, Renmin South Road 16, Chengdu 610041, China; scu_gaojiaqi@163.com (J.G.); liujin1231@stu.scu.edu.cn (J.L.); xingzhao@scu.edu.cn (X.Z.); 2 Department of International Medical Center/Ward of General Practice, West China Hospital, Sichuan University, Renmin South Road 16, Chengdu 610041, China

**Keywords:** ABO blood types, genome-wide association study, mendelian randomization, Chinese cohort

## Abstract

ABO blood system is an inborn trait determined by the *ABO* gene. The genetic-phenotypic mechanism underneath the four mutually exclusive and collectively exhaustive types of O, A, B and AB could theoretically be elucidated. However, genetic polymorphisms in the human populations render the link elusive, and importantly, past studies using genetically determined rather than biochemically determined ABO types were not and could not be evaluated for the inference errors. Upon both blood-typing and genotyping a cohort of 1008 people of the Han Chinese population, we conducted a genome-wide association study in parallel with both binomial and multinomial log-linear models. Significant genetic variants are all mapped to the *ABO* gene, and are quantitatively evaluated for binary and multi-class classification performances. Three single nucleotide polymorphisms of *rs8176719*, *rs635634* and *rs7030248* would together be sufficient to establish a multinomial predictive model that achieves high accuracy (0.98) and F1 scores (micro 0.99 and macro 0.97). Using the set of identified ABO-associated genetic variants as instrumental variables, we demonstrate the application in causal analysis by Mendelian randomization (MR) studies on blood pressures (one-sample MR) and severe COVID-19 with respiratory failure (two-sample MR).

## 1. Introduction

Discovered earliest in 1901 by Karl Landsteiner, the ABO system classifies human blood into exclusive types of O, A, B and AB based on the presence or absence of specific A and B antigens on the surface of red blood cells [1]. The key determinant, the *ABO* gene, consists of seven exons located on chromosome 9q34.2 and encodes a glycosyltransferase that catalyzes the transfer of carbohydrates to the H antigen to form the antigenic structures of the ABO blood groups [2]. The amino acid sequences encoded by the A and B alleles of the *ABO* gene differ minimally, and they catalyze the transfer of different carbohydrates (N-acetyl-galactosamine or galactose) to form the A or B antigen, respectively; in comparison, the *ABO* gene in individuals with blood type O does not produce A or B antigens due to a single-base nonsense deletion [3]. The *ABO* gene displays a substantial spectrum of single nucleotide polymorphisms (SNPs): over 100 unique alleles have been curated for the *ABO* gene in the Leiden Open Variation Database [4]. While biochemical assessment of ABO blood types is a routine procedure in healthcare laboratories, it was rarely conducted in scientific research on humans for which ABO blood types are commonly genetically inferred from pre-defined SNP allele combinations [5]. For example, UK Biobank did not determine ABO blood types of the blood samples from cohort participants, and consequently, all studies using UK Biobank data must genetically determine the ABO blood types [5]. However, the errors introduced by the genetic inferences instead of the golden standard of biochemical blood-typing were rarely evaluated, and could be significant given the large sizes of cohorts.

The *ABO* gene is expressed not only in red blood cells but also in body fluids and tissues surfaces (including epithelial cells, sensory neurons, endothelial cells, kidney, heart, pancreas, platelets and lungs), suggesting the extension of the ABO system in clinical importance beyond immunohematology and transfusion/transplantation medicine [6,7]. The Pancreatic Cancer Cohort Consortium (PanScan) reported that the most significant variants associated with pancreatic cancer risk were mapped to the *ABO* locus [8]. A genetic variant of *rs579459* in *ABO* was associated with the risk of coronary artery disease [9]. Notably, COVID-19 is a novel type of coronavirus first appearing in late 2019 and causing an ongoing global pandemic that has led to over 107 million cases and 2.3 million deaths by February 2021 [10], ranking the COVID-19-related pneumonia as one of the most devastating infectious diseases in the history of public health [11]. Several observational studies discovered a preliminary association between ABO blood types and COVID-19 susceptibility [12,13,14]. Interestingly, in the first GWAS on COVID-19 carried out in a European cohort (Italian and Spanish), one of the two genome-wide significant SNP hits is *rs657152* at the *ABO* gene (the other is *rs11385942* at the *LZTFL1* gene) [15]. The underlying mechanism between ABO blood types and COVID-19 susceptibility remains absent, and it is still highly debated if such association could be causal.

Genome-wide association study (GWAS) is a high-throughput method sequencing genomic variants and mapping SNPs associated with the phenotype-of-interest [16]. Hit SNPs could be influenced by the genetic background of the population so the GWAS-revealed ABO-disease link may even be population or ethnicity specific [17,18]. In addition, the world has long been disproportionally represented in population diversity with regard to GWAS cohorts, limiting the scientific applicability of the relevant discoveries [19,20]. In this study, we aim to in parallel blood-type and genotype a cohort, and thus be able to conduct a GWAS using ABO blood types as the phenotype-of-interest. In addition, we focus on the selection of a Han Chinese cohort to identify the set of genetic variants that could be applied for the accurate genetic determination of ABO blood types in the most populous ethnicity on earth. Finally, we demonstrate the application of selecting ABO-associated SNPs as instrument variables in Mendelian randomization (MR) studies for causal inference between the exposure (ABO blood types) and the outcomes (traits or diseases of interest). The results and discoveries not only elucidate the genetic link behind the ABO blood system, but also contribute to the understanding of the association of ABO blood types with an increasing number of traits and diseases.

## 2. Materials and Methods

### 2.1. Study Design and Ethics

A group of healthy adult residents of the Han ethnicity in China were recruited as a cohort based on multi-stage sampling from the China Multi-Ethnic Cohort (CMEC) [21]. For GWAS, traits or phenotypes in the participants were subjected to association analysis with genomic polymorphisms.

For this study, serological testing of the ABO blood system was conducted for each individual by means of a routine blood test: 2 mL of blood sample were collected, transported cold back to a certified clinical laboratory within 24 h and assayed for agglutination using antisera reagents to identify blood group antigens (ABO blood types as the outcome-of-interest). In parallel, genotyping of single nucleotide polymorphisms (SNPs) was carried out for each individual: 1–2 mL of saliva sample were collected, transported back to a certified genetic laboratory within 24 h, subjected to genomic DNA purification and finally genotyped on a high-throughput multi-channel platform (Thermo Fisher Scientific, Waltham, MA, USA) using Affymetrix Axiom Precision Medicine Array chips (genomic SNPs as the predictors). Other related health and personal information of the participants was collected on site through face-to-face surveys by trained staff (potential confounding factors as the covariates). The methods were carried out in accordance with the relevant guidelines and regulations.

For one-sample MR analysis, individuals’ blood pressures (systolic and diastolic) were measured by certified medical staff; for two-sample MR analysis, genomic associations with COVID-19 phenotype were retrieved from published literature [15].

The study design and procedure were reviewed and approved by the Research Ethics Committee at West China School of Public Health of Sichuan University. All participants in the cohort were informed of the research design, and have signed the Informed Consent Form. The phenotype and genetic data were subjected to anonymization for the protection of personal privacy. In observance of the Regulatory Articles of Human Genetic Resources of P.R. China, population-based summary statistics are reported for GWAS but no individual’s raw genotypes were published.

### 2.2. Data Analysis

A dataset of 1008 individuals with their blood typing of ABO system, genotyping of 733,907 SNPs and related personal information (sex, age, etc.) was assembled by the completion of routine blood tests, lab genotyping experiments and on-site surveys. SNPs on the autosomes were used in association analysis. The raw genetic dataset was pre-processed for quality checks at both sample and SNP levels. For GWAS, two respective models were fit: (1) binomial logistic regression model with the ABO blood types dichotomized into a binary phenotype (e.g., O vs. non-O), and (2) multinomial log-linear model with the ABO blood types treated as a quaternary phenotype (O, A, B, AB). For two-allele SNP settings, the major allele was defined as the reference allele, and the minor allele was thus selected as the effect allele. Locus genotypes were coded as the copies of minor allele, accordingly assuming an additive effect. The top ten principal components (PCs) from principal component analysis (PCA) on SNPs in linkage equilibrium were included in modeling for the adjustment of any remaining genetic substructures. In accordance with common GWAS practice, sex and age were included as covariates in modeling, but in the case of ABO blood types, inclusion or omission of sex and age would not lead to significant differences because the ABO blood types for individuals are determined from birth (do not change as the age grows), and are inherited in an autosomal rather than sex-dependent manner (no association with the sex). We include sex and age in the linear models as covariates to match the general practice of published GWAS for many diseases or traits (for which sex or age is actually associated with), but in parallel, we have conducted GWAS analysis without adjusting sex and age, and the top hit SNPs remain the same (the no-sex-and-age GWAS results are not included in this manuscript to avoid redundancy, but in case they are useful, are available to other researchers by contacting Mengqiao Wang). With a commonly used significance level of 0.05 adjusted by one million (the number of SNPs assayed is 733,907), a conservative Bonferroni corrected threshold of 5 × 10^−8^ (−log_10_(*p*) of 7.3) was used exclusively for screening hit SNPs (see Supplemental Materials Tables S3–S9 for summary statistics). In binary scenarios of ABO blood types (Tables S3–S6), the selection of SNPs for predictive modeling is an iterative process of (1) screening for strong OR with significant *p* value, and (2) avoidance of linkage disequilibrium (*r*^2^ < 0.8). In these tables, # suffix in SNP annotates the subset of hit variants selected and to be evaluated for binary classification performances in Table 1. For example, *rs529565* is the top ranked SNP for O vs. non-O and selected for binary classification evaluation (thus annotated with the suffix as *rs529565#*. With linkage analysis from Figure S5A, those hit variants with high linkage (*r*^2^ ≥ 0.8) to *rs529565* would not be selected for binary classification evaluation (thus no # suffix: *rs657152* with *r*^2^ = 0.94 to *rs529565*). Iterating the above analysis for all hit SNPs leads to the selection of # suffix genetic variants to be evaluated (Table 1). The inverse-variance-weighted (IVW) and weighted median methods were applied for Mendelian randomization analysis.

Statistical analysis and data visualization were conducted using version 4.0.0 of the R statistical environment (R core team, 2020) and PLINK 1.07 [22]. Single-machine parallel computing of GWAS was run on a Windows platform with an Intel CPU (32G RAM, 3.6 GHz, 8 cores).

### 2.3. Analytical Resources

The core codes of binomial and multinomial models used in this study are available from the following public GitHub repository: GWAS-ABO https://github.com/westchinabiomedicaldatascience-Wang-lab/GWAS-ABO (accessed on 6 November 2020). The authors have provided the relevant information (allele frequency, *p* value, OR, etc.) for hit SNPs in Supplemental Materials.

## 3. Results

### 3.1. A GWAS on ABO Blood Types in a Chinese Population

An initial cohort of 1008 individuals fully participated in blood-typing, genotyping and on-site surveys. Upon sequential steps of data pre-processing, a final dataset of 921 independent individuals was ready for GWAS analysis (87 individuals dropped: 7 for failure in genotyping, 1 for low sample call rate and 79 for genome-wide identity-by-descent pairwise relatedness). The cohort was summarized for baseline characteristics that are comparable across the four levels of ABO blood system (Table S1). In addition, with robustness in sampling, the distribution of ABO blood types in this cohort is similar to other Chinese or Asian populations (Table S2). Upon blood-typing and genotyping, a GWAS was conducted to screen and map genetic variants key to the determination of ABO blood types (Figure S1). For the outcome, two analytical methods are applied in parallel: (1) dichotomize ABO blood types into four sets of binary outcomes (e.g., O vs. non-O, with A, B and AB individuals combined into the non-O group), and (2) treat ABO blood types with respect to their innate status as a quaternary outcome (four levels of O, A, B and AB).

### 3.2. GWAS on ABO Blood Types Based on Binomial Models

For the binomial models, all hit SNPs are mapped exclusively to the *ABO* gene and its nearby regions (Tables S3–S6, Figures S2–S4), validating ABO blood system as a classical Mendelian phenotype determined by a single gene. Certain hit SNPs are in strong linkage disequilibrium (LD) (Figure S5) and display similar estimates for both the *p* value and odds ratio (Tables S3–S6). Accordingly, based on LD screening (threshold of *r*^2^ < 0.8) to avoid the most notable redundant variants, a subset of hit SNPs are selected in each binomial model (annotated with # symbols in Tables S3–S6) and respectively evaluated for binary classification performances calculated with the baseline type prevalence as the threshold, and as well as the area under the ROC curve (AUC) defined by a series of thresholds (Table 1, 16 SNPs in O vs. non-O, 12 SNPs in A vs. non-A, 8 SNPs in B vs. non-B, 6 SNPs in AB vs. non-AB): *rs529565* alone is almost sufficient to differentiate O vs. non-O groups; *rs635634* displays the best performance in A vs. non-A groups, boosted by the further inclusion of *rs8176719*; similarly, *rs7030248* excels in the comparison of B vs. non-B groups, with additional support from the inclusion of *rs635634*, and *rs687289* stands out as the key variant for differentiating AB vs. non-AB groups.

Therefore, the most significant hit SNP selected for each blood type in the binomial models is *rs529565* for O vs. non-O, *rs635634* for A vs. non-A, *rs7030248* for B. vs. non-B and *rs687289* for AB vs. non-AB. However, among these four selected SNPs, the pair of *rs529565* and *rs687289* are in high LD (*r*^2^ = 0.97, Figure S5B), so a common SNP of *rs8176719* in LD with both of these SNPs could be selected to replace them: *rs529565* is in high LD with *rs8176719* (*r*^2^ = 0.95, Figure S5B), and rs687289 is in high LD with *rs8176719* (*r*^2^ = 0.98, Figure S5B). In addition, *rs8176719* is not in high LD with the other two selected SNPs of *rs635634* (*r*^2^ = 0.35, Figure S5B) and *rs7030248* (*r*^2^ = 0.09, Figure S5B), so these three SNPs present a group of independent variants associated with the ABO blood types and would be later evaluated for multi-class classification performances.

### 3.3. GWAS on ABO Blood Types Based on Multinomial Models

**Figure 1:**
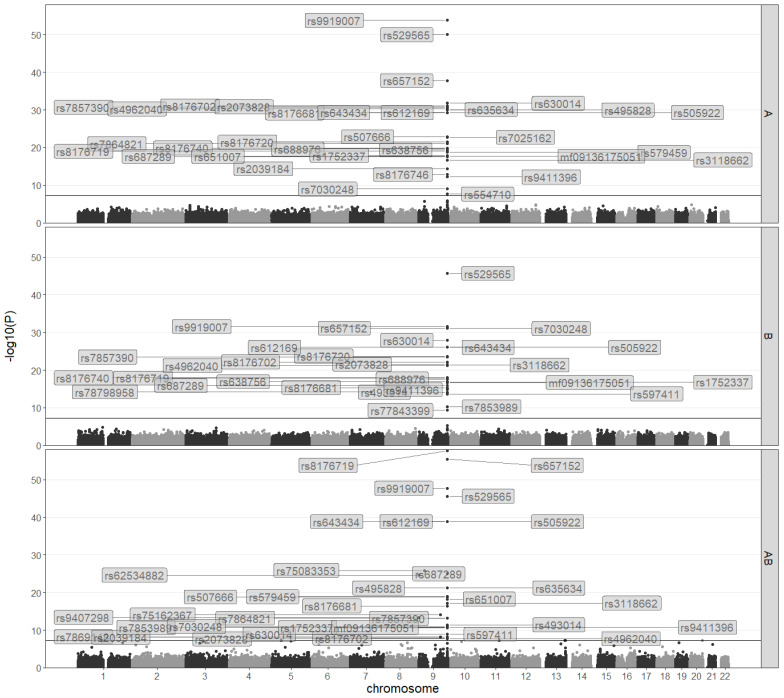
Manhattan plots for the quaternary (4-level, reference level of type O) outcomes of A vs. O (upper), B vs. O (middle) and AB vs. O (lower) in the cohort. Genome-wide hit SNPs (above the horizontal line) are annotated.

**Figure 2:**
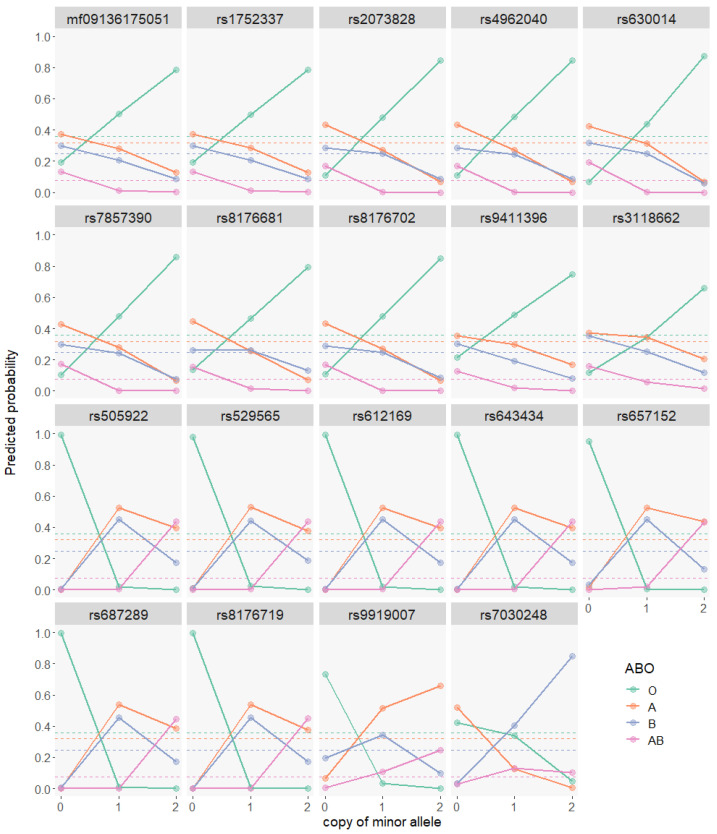
Predicted probability of individual’s ABO blood types based on 19 hit SNPs shared among the multinomial models. The dashed horizontal lines annotate the prevalence of respective ABO blood types in the cohort.

Dichotomizing ABO blood types into a binary phenotype is a common practice in previous studies, but such an operation leads to the loss of information and would reduce statistical power. In addition, predicted probabilities for each blood type from the four binomial models do not add up to one, leading to the difficulty in appropriately assigning un-blood-typed but genotyped individuals to a specific predicted blood type. To overcome such issues, we treat ABO blood types as a quaternary phenotype (with type O defined as the reference level), perform a multinomial analysis for GWAS and consistently uncover associated variants mapped exclusively to the *ABO* gene and nearby regions (Tables S7–S9, Figure 1 and Figure S6). There are in total 19 hit SNPs shared across all three multinomial models (Tables S7–S9), and some of these genetic variants display high LD (Figure S7). Predictive probability for an individual’s blood type based on personal genotypes of these 19 commonly significant hit SNPs reveals four major patterns (Figure 2): (1) 10 SNPs are associated with the likelihood of type O; (2) 7 SNPs (including *rs8176719*) are associated with higher probability of type A and type B for single copy of minor alleles but of type AB for dual copies; (3) *rs9919007* is associated with higher probability of type B for single copy but of type A and type AB for dual copies; (4) *rs7030248* is predominantly associated with type B. These distinct patterns reveal hit SNPs associated with the respective type of the ABO blood system.

**Figure 3:**
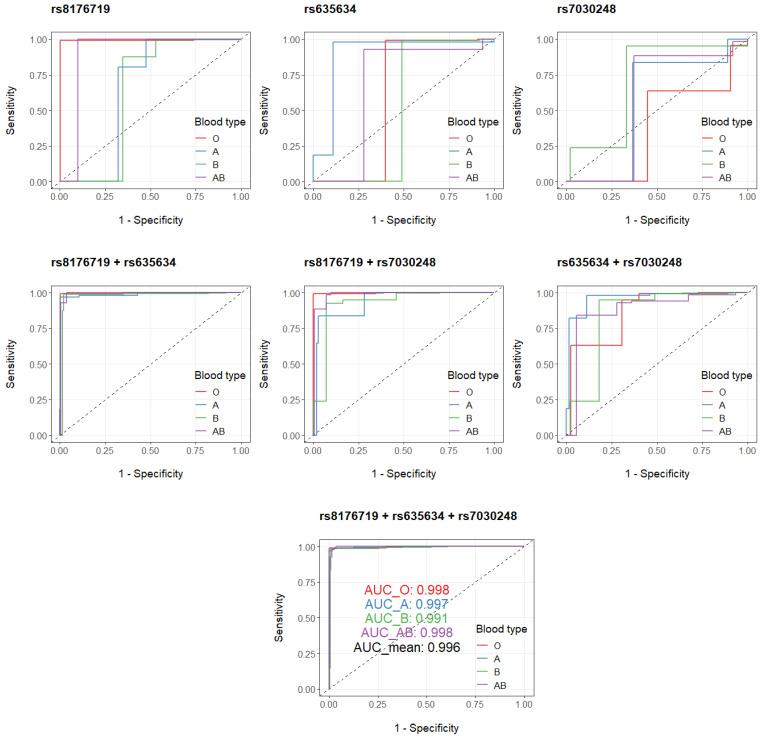
By-blood-type ROC plots for *rs8176719*, *rs635634*, *rs7030248* and their combinations. AUC: area under the curve.

**Figure 4:**
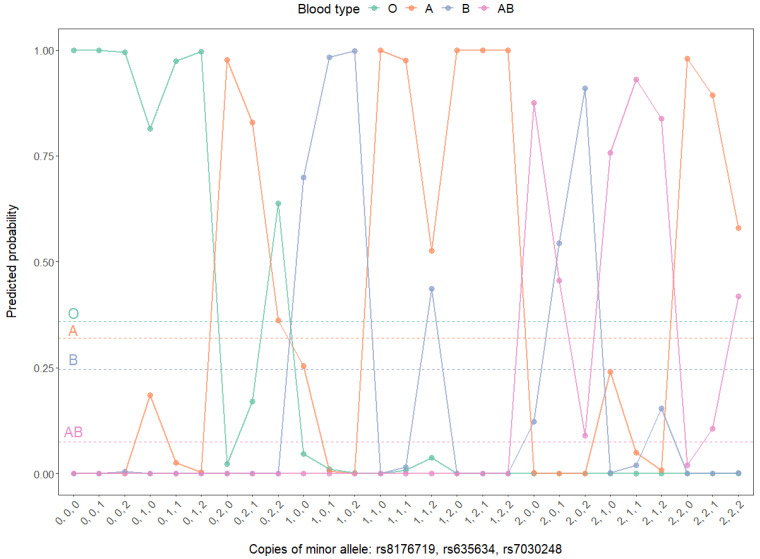
Predicted probabilities of ABO blood types based on the combined set of *rs8176719*, *rs635634* and *rs7030248* in the multinomial model. The dashed horizontal lines annotate the prevalence of respective ABO blood types in the cohort. Note: due to the limited sample size and the factor of minor allele frequency, not all allele combinations (3^3^ = 27) of the three SNPs are represented in the cohort (the 16 combinations represented are tabulated in Table S10; the other 11 combinations are not present in the cohort, so the prediction of ABO blood types for these genotype sets could not be evaluated for prediction performances).

The set of three independent hit SNPs identified from binomial models are well represented in the hit SNPs from multinomial models: *rs8176719* and *rs7030248* are among the 19 common hit SNPs (Tables S7–S9); *rs635634* associates with A vs. O (Table S7) and AB vs. O (Table S9). With overall parameters such as accuracy, F1 micro and F1 macro calculated for multi-class performances and classical binary parameters evaluated for by-blood-type binary performances, the genetic variants of *rs8176719*, *rs635634* and *rs7030248* are evaluated both singularly and combinatorically in the multinomial models (Figure S8). These three SNPs display a synergistic effect in that additive combination sequentially improves both multi-class and binary classification performances. With all three SNPs combined, almost perfect prediction of ABO blood types could be achieved in the multinomial models for the cohort (Figure S8C). Only 19 out of a total of 916 individuals are wrongly assigned (Table S10), and for these 19 off-target predictions, a partial on-target effect is present (Table S11) since there is no mispredictions of type O for type AB or vice versa, considering these two types are biochemically more distinct among all four types. Effectiveness and robustness in predicting ABO blood types with the set of three SNPs is also validated by the sequential boost observed in both precision–recall curves (Figure S9) and ROC curves (Figure 3). Indeed, the “*rs8176719* + *rs635634* + *rs7030248*” multinomial model leads to well-separated predicted probabilities for ABO blood types in all possible scenarios (Figure 4).

### 3.4. Causal Inference by Mendelian Randomization

**Figure 5:**
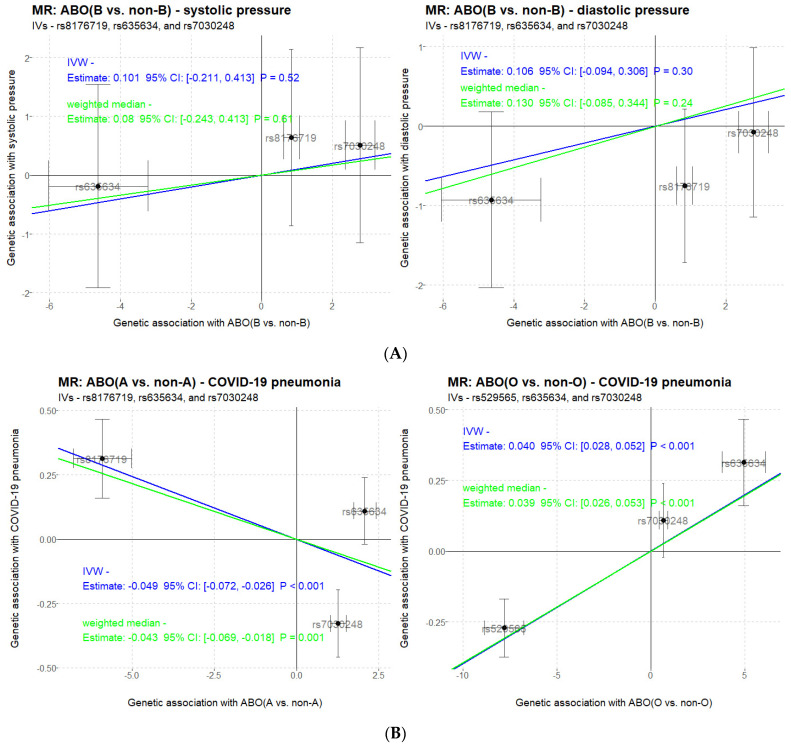
Mendelian randomization analysis between ABO blood types (exposure) and phenotypes-of-interest (outcome). (**A**) One-sample MR of ABO-blood pressures; (**B**) two-sample MR of ABO-severe COVID-19 with respiratory failure.

It was reported that type B is more susceptible to hypertension [23]. We measured the systolic and diastolic pressures of the cohort, and using the validated set of three ABO-associated SNPs (*rs8176719*, *rs635634* and *rs7030248*) as instrumental variables in one-sample MR, two methods of inverse-variance weighted (IVW) and weighted median argue against a potential causal relationship between the exposure of ABO blood types and the outcome of blood pressures in the Han Chinese population (Figure 5A).

The COVID-19 GWAS project not only discovers ABO-mapped *rs657152* as a hit SNP but also validates the association using inferred blood types for a higher risk in blood group A (OR of 1.45) and a protective effect in blood group O (OR of 0.65) [15]. With estimates of the instrumental variables selected for ABO blood types retrieved from this study (ethnicity of Han Chinese) and for severe COVID-19 with respiratory failure retrieved from published literature (ethnicities of Italian and Spanish), we conduct a two-sample MR in which both IVW and weighted median methods strongly support a causal link (*p* < 0.001, Figure 5B) in the A vs. non-A as well as O vs. non-O contrasts with differential COVID-19 susceptibility. However, the direction of MR relationship in this study (A type is less susceptible and O type is more susceptible to the risk of severe COVID-19 with respiratory failure) is not consistent with the direction reported in the COVID-19 GWAS study [15].

## 4. Discussion

Our extensive characterization of the ABO genomics validates the ABO blood system as a classical Mendelian phenotype determined exclusively by a single *ABO* gene. Therefore, perfect prediction of ABO blood types based on genetic variants is theoretically possible. We demonstrate that while dichotomizing ABO blood system into a set of binary phenotypes is capable of identifying key genetic variants, treating the phenotype as a quaternary trait and applying a multinomial model would confer higher statistical power and simultaneously evaluate the probabilities of an individual for all four types. A key contribution of our study lies in the simultaneous genotyping and blood-typing of the cohort members so the genetics-based model could be evaluated and optimized for predictive performances. In contrast, past studies subjectively infer blood types from genotypes without quantifying the potential error rate. To make the practice more error-prone, past genetic association studies on ABO blood types generally use individuals’ self-reported rather than biochemically determined ABO blood types, raising concerns for the identity and strength of the hit SNPs used for ABO inference.

In this study, a predictive model based on *rs8176719*, *rs635634* and *rs7030248* leads to strong performances in multi-class classification, suggesting these three SNPs combined would be sufficient to explain a major proportion of the diversity in the ABO blood system within the Han Chinese population. Given that the allele frequencies of the hit SNPs are comparable but still disparate among different ethnicities (Figure S10), the predictive models from this Han Chinese cohort should be applicable to other ethnicities with potentially limited sacrifice of forecast accuracy. Nevertheless, there is still a small portion of individuals mistakenly assigned using the three-SNP-based predictive model, suggesting that a complete understanding of the underlying genetic-phenotypic link between *ABO* polymorphisms and ABO blood system would in the future require better-informed genotyping (e.g., whole gene sequencing is preferred for both full coverage of all possible genetic variants and as well for the haplotypes). Such efforts combined with larger sample size and populations of different ethnicities should deepen our understanding of the ABO genomics and also optimize a predictive model that precisely or even perfectly determines blood types solely based on genetics.

An interesting observation from GWAS is that the top hit SNPs with the lowest *p* value or highest odds ratio are not necessarily those missense variants that confer structural changes to the glycosyltransferase encoded by the *ABO* gene. This raises an unverified issue if the ABO blood system could be solely determined by the exons of the *ABO* gene, or would be dependent on genetic variants both in the exons and introns. Undoubtedly, we only genotyped a subset of all possible missense genetic variants, and there also exists high LD in the region. Therefore, it would be meaningful for future research to conduct whole-gene-sequencing-based association studies to appropriately resolve this issue.

The involvement of the ABO blood system in biology and health has been ever increasing. Historically, ABO blood-typing is widely applied in paternity testing in an “exclusion” basis before being replaced by the genotyping-based “confirmation” methods [24]. ABO blood system is also essential in daily medical operations of blood donation/transfusion and organ transplantation [25,26]. There is even an ABO personality theory popular in certain regions of the world [27,28]. The fact that *ABO* variants are discovered in large-scale GWAS for association with an increasing number of traits and diseases naturally leads to the necessity to differentiate causality from non-causal association. MR is a fast-developing field to address such causal inferences, but certain key concerns exist. First, the exposure of ABO blood types is treated as a binary variable, which is uncommon but not impossible in MR [29]. Second, the assumptions of MR are essential for the validity of causal interpretation, and regarding ABO blood types, an assumption that might fail to meet is that the *ABO* gene or variants could be pleiotropic. Finally, there is always a potential but hard-to-evaluate issue regarding ethnicity confounding in two-sample MRs, which could have profound consequences when the *p* value is marginally significant or insignificant (e.g., around 0.05). For the above reasons, the causal inference results from our two-sample MR study between ABO and COVID-19 should be viewed as preliminary rather than conclusive.

## Figures and Tables

**Table 1:** Classification performance of selected SNPs in GWAS for binary ABO blood types.

SNP	Accuracy	Sensitivity	Specificity	PPV	NPV	F1	AUC
***O vs. non-O***							
*rs2039184*	0.64	0.49	0.72	0.5	0.72	0.49	0.61
*rs76321958*	0.63	0.38	0.77	0.48	0.69	0.43	0.58
*rs7864821*	0.67	0.55	0.73	0.54	0.75	0.55	0.64
*rs9919007*	0.86	0.96	0.81	0.73	0.97	0.83	0.88
*rs8176740*	0.68	0.68	0.68	0.54	0.79	0.6	0.68
*rs7853989*	0.69	0.99	0.52	0.54	0.99	0.7	0.75
*rs4962040*	0.67	0.82	0.58	0.53	0.85	0.64	0.7
*rs529565*	0.98	0.97	0.99	0.98	0.98	0.97	0.98
*rs630014*	0.65	0.88	0.53	0.5	0.89	0.64	0.7
*rs651007*	0.75	0.99	0.61	0.59	0.99	0.74	0.8
*rs7030248*	0.58	0.64	0.55	0.44	0.73	0.52	0.59
*rs1752337*	0.66	0.69	0.65	0.52	0.79	0.6	0.67
*rs493014*	0.49	0.94	0.25	0.41	0.87	0.57	0.59
*rs9411396*	0.65	0.66	0.65	0.51	0.77	0.57	0.65
*rs12763*	0.59	0.58	0.6	0.45	0.72	0.51	0.59
*rs3118662*	0.72	0.43	0.88	0.67	0.73	0.52	0.66
full set	0.99	0.99	0.99	0.99	1	0.99	0.99
***A vs. non-A***							
*rs2039184*	0.54	0.78	0.42	0.39	0.8	0.52	0.6
*rs7864821*	0.56	0.8	0.45	0.4	0.82	0.54	0.62
*rs9919007*	0.74	0.93	0.66	0.56	0.95	0.7	0.79
*rs7857390*	0.6	0.56	0.62	0.41	0.75	0.47	0.59
*rs7853989*	0.63	0.98	0.47	0.46	0.99	0.62	0.73
*rs8176720*	0.77	0.62	0.83	0.63	0.83	0.63	0.73
*rs8176719*	0.68	1	0.52	0.5	1	0.66	0.76
*rs630014*	0.6	0.47	0.66	0.4	0.73	0.43	0.57
*rs635634*	0.92	0.98	0.89	0.81	0.99	0.88	0.93
*rs7030248*	0.7	0.84	0.64	0.52	0.89	0.64	0.74
*rs7025162*	0.6	0.9	0.47	0.44	0.91	0.59	0.68
*rs13289928*	0.59	0.61	0.59	0.41	0.76	0.49	0.6
*rs635634* + *rs8176719*	0.99	0.97	0.99	0.99	0.99	0.98	0.98
full set	1	1	1	1	1	1	1
***B vs. non-B***							
*rs77843399*	0.78	0.22	0.96	0.65	0.79	0.32	0.59
*rs8176720*	0.81	0.47	0.92	0.65	0.84	0.54	0.69
*rs8176719*	0.6	1	0.47	0.38	1	0.55	0.73
*rs635634*	0.63	0.99	0.51	0.4	0.99	0.57	0.75
*rs7030248*	0.74	0.95	0.67	0.48	0.98	0.64	0.81
*rs7025162*	0.69	0.58	0.73	0.41	0.84	0.48	0.65
*rs493014*	0.75	0.36	0.88	0.49	0.81	0.41	0.62
*rs3118662*	0.67	0.37	0.77	0.34	0.79	0.35	0.57
*rs7030248* + *rs635634*	0.85	0.95	0.82	0.63	0.98	0.76	0.88
full set	0.99	0.99	0.99	0.96	1	0.97	0.99
***AB vs. non-AB***							
*rs9919007*	0.54	0.96	0.5	0.14	0.99	0.24	0.73
*rs8176746*	0.75	1	0.73	0.23	1	0.38	0.87
*rs687289*	0.91	0.99	0.9	0.44	1	0.61	0.94
*rs8176681*	0.63	0.9	0.6	0.16	0.99	0.27	0.75
*rs507666*	0.68	0.97	0.65	0.19	1	0.31	0.81
*rs3118662*	0.74	0.55	0.76	0.16	0.95	0.24	0.66
full set	0.91	0.99	0.9	0.46	1	0.62	0.94

Note: PPV/NPV—positive/negative predictive value; F1—F1 score; AUC—area under the ROC curve.

## Data Availability

The core codes of binomial and multinomial models used in this study are available from the following public GitHub repository: GWAS-ABO https://github.com/westchinabiomedicaldatascience-Wang-lab/GWAS-ABO (accessed on 6 November 2020). Relevant information of GWAS (allele frequency, *p* value, OR, etc.) for hit SNPs are provided in the Supplemental Materials.

## Supplementary Materials

The following are available online at https://www.mdpi.com/article/10.3390/genes12070959/s1, Figure S1. Schematic diagram for the study design of GWAS on ABO blood types in a Chinese population. Figure S2. Manhattan plots for the binary (2-level) outcomes: O vs. non-O (A), A vs. non-A (B), B vs. non-B (C) and AB vs. non-AB (D) in the cohort. Figure S3. Quantile-quantile plots for the binary (2-level) outcomes: O vs. non-O (A), A vs. non-A (B), B vs. non-B (C) and AB vs. non-AB (D) in the cohort. Figure S4. Regional association plots for the binary (2-level) outcomes: O vs. non-O (A), A vs. non-A (B), B vs. non-B (C) and AB vs. non-AB (D) in the cohort. Figure S5. Linkage analysis (r2) between ABO-associated hit SNPs for the binary (2-level) outcomes: O vs. non-O (A), A vs. non-A (B), B vs. non-B (C) and AB vs. non-AB (D) in the Chinese (CHB + CHS) from the 1000 Genomes. Figure S6. Regional association plots for the quaternary (4-level, reference level of type O) outcomes of A vs. O (upper), B vs. O (middle) and AB vs. O (lower) in the cohort. Figure S7. Linkage analysis (r2) between ABO-associated hit SNPs for the quaternary (4-level, reference level of type O) outcomes: A vs. O (A), B vs. O (B) and AB vs. O (C) in the Chinese (CHB + CHS) from the 1000 Genomes. Figure S8. Confusion matrix (left) and classification performance parameters (right) for the prediction of ABO blood types based on rs8176719, rs635634, rs7030248 (A), and their combinations (B and C). Figure S9. By-blood-type precision-recall plots for rs8176719, rs635634, rs7030248, and their combinations. Figure S10. Allele frequency of common hit SNPs in the multinomial models from this study among the globe and five super-ancestries from the 1000 Genomes. Table S1. Summary of the general and by-type baseline characteristics. Table S2. Distribution of ABO blood types across Asian ethnicities. Table S3. Summary of GWAS on the binary trait of O vs. non-O blood types. Table S4. Summary of GWAS on the binary trait of A vs. non-A blood types. Table S5. Summary of GWAS on the binary trait of B vs. non-B blood types. Table S6. Summary of GWAS on the binary trait of AB vs. non-AB blood types. Table S7. Summary of GWAS on the quaternary trait of A vs. O blood types. Table S8. Summary of GWAS on the quaternary trait of B vs. O blood types. Table S9. Summary of GWAS on the quaternary trait of AB vs. O blood types. Table S10. Frequency summary of individuals with the defined set of SNPs (*n* = 916). Table S11. Summary of individuals with wrongly predicted ABO blood types (*n* = 19).
