# Supplementary material for: Genomic Association vs. Serological Determination of ABO Blood Types in a Chinese Cohort, with Application in Mendelian Randomization
Source: Genes (Basel). 2021 Jun 24;12(7):959. doi: 10.3390/genes12070959 (PMC8306414; doi:10.3390/genes12070959)
Supplement: Supplementary file 1 [file genes-12-00959-s001.zip › genes-1221121-supplementary.pdf]

Supplemental Materials

- Supplemental figures (10)
- Supplemental tables (11)

Supplementary figures

Figure S1

Schematic diagram for the study design of GWAS on ABO blood types in a Chinese population

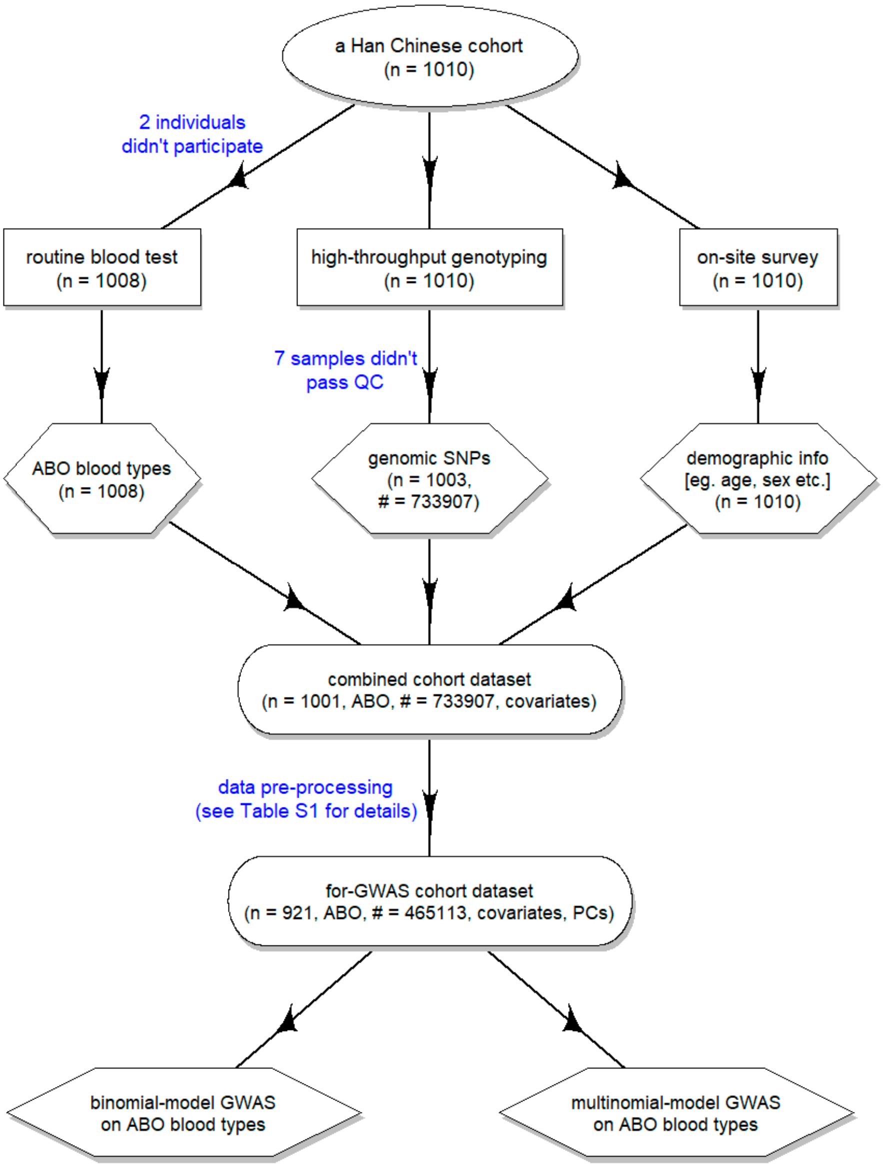

Figure S2

A.

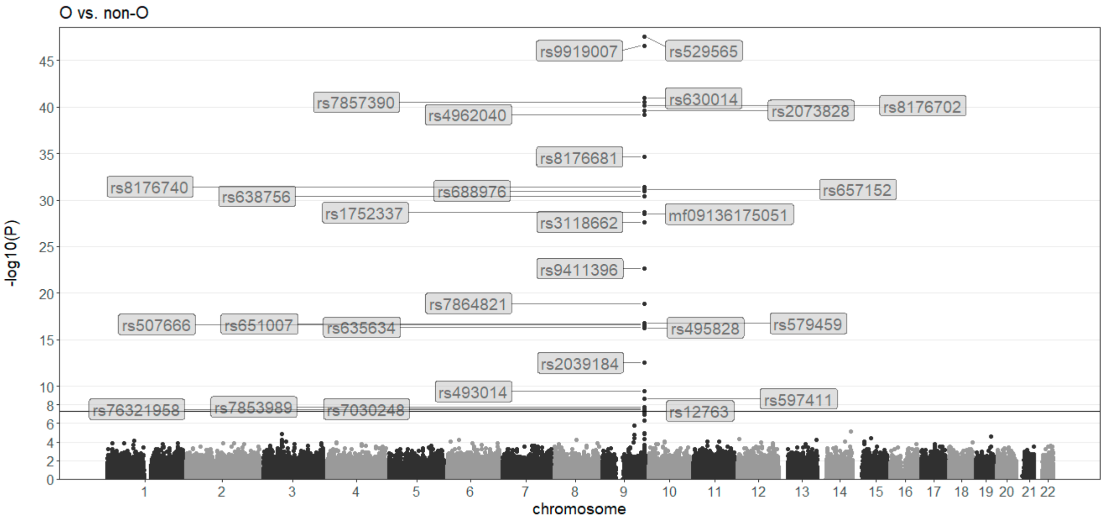
O vs. non-O
-log10(P)
chromosome
rs9919007
rs529565
rs7857390
rs4962040
rs630014
rs2073828
rs8176702
rs8176681
rs8176740
rs638756
rs688976
rs657152
rs1752337
rs3118662
mf09136175051
rs9411396
rs7864821
rs507666
rs651007
rs635634
rs495828
rs579459
rs2039184
rs493014
rs76321958
rs7853989
rs7030248
rs12763
rs597411

B.

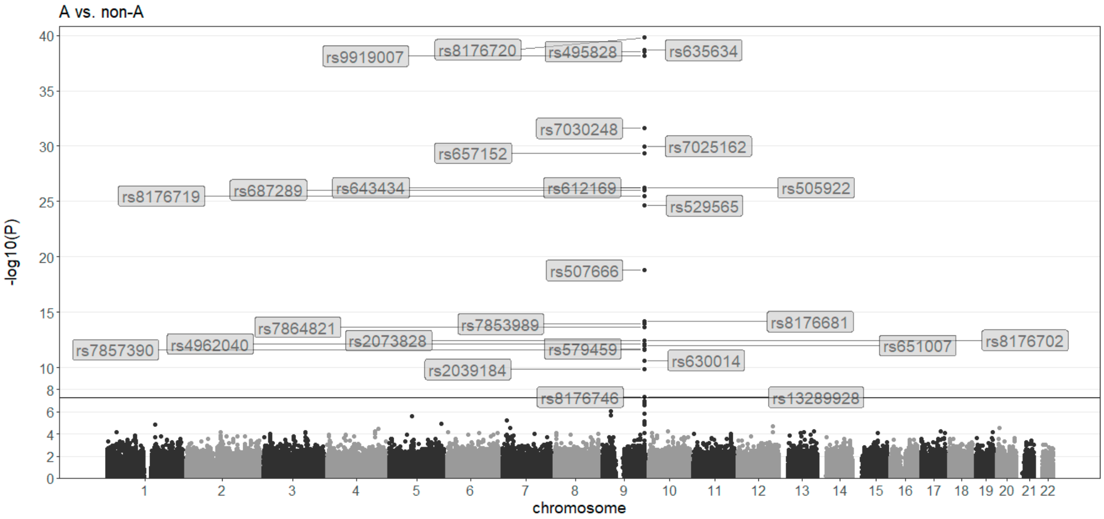
A vs. non-A
-log10(P)
chromosome
rs9919007
rs8176720
rs495828
rs635634
rs7030248
rs657152
rs7025162
rs8176719
rs687289
rs643434
rs612169
rs505922
rs529565
rs507666
rs7864821
rs7853989
rs8176681
rs7857390
rs4962040
rs2073828
rs579459
rs651007
rs8176702
rs2039184
rs630014
rs8176746
rs13289928

C.

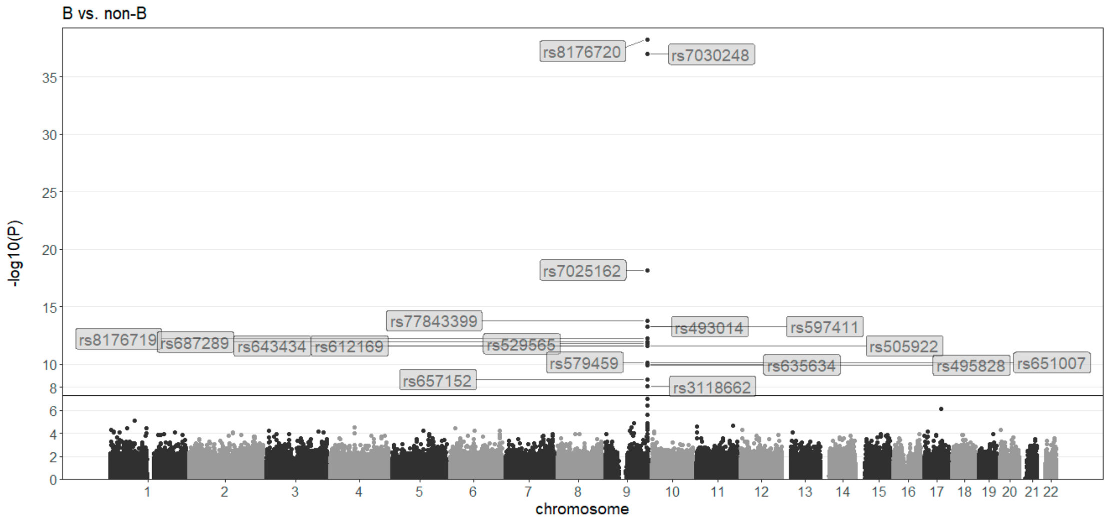
B vs. non-B
-log10(P)
chromosome
rs8176720
rs7030248
rs7025162
rs77843399
rs493014
rs597411
rs8176719
rs687289
rs643434
rs612169
rs529565
rs505922
rs579459
rs635634
rs495828
rs651007
rs657152
rs3118662

D.

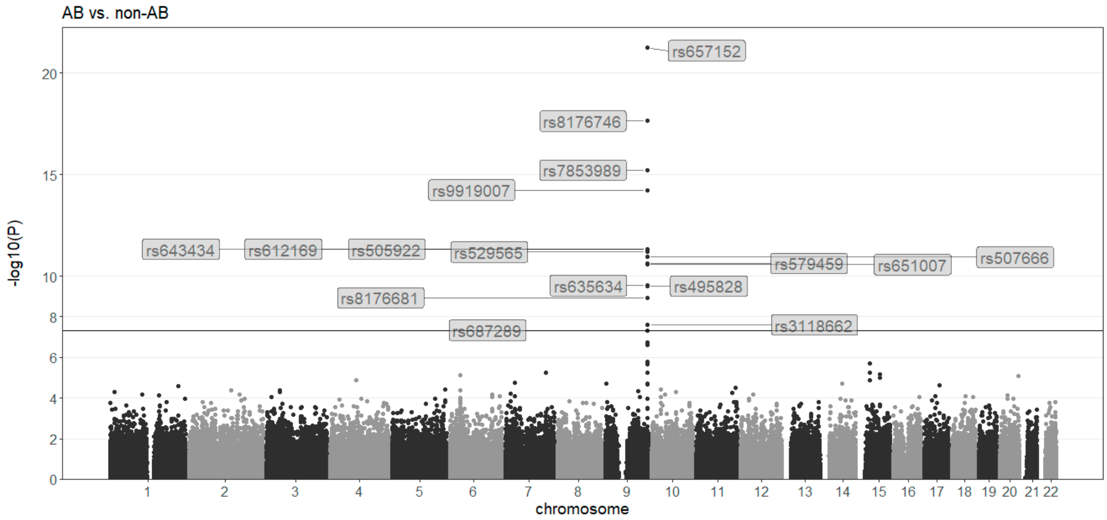
AB vs. non-AB
-log10(P)
chromosome
rs657152
rs8176746
rs7853989
rs9919007
rs643434
rs612169
rs505922
rs529565
rs579459
rs651007
rs507666
rs635634
rs495828
rs8176681
rs3118662
rs687289

Figure S3

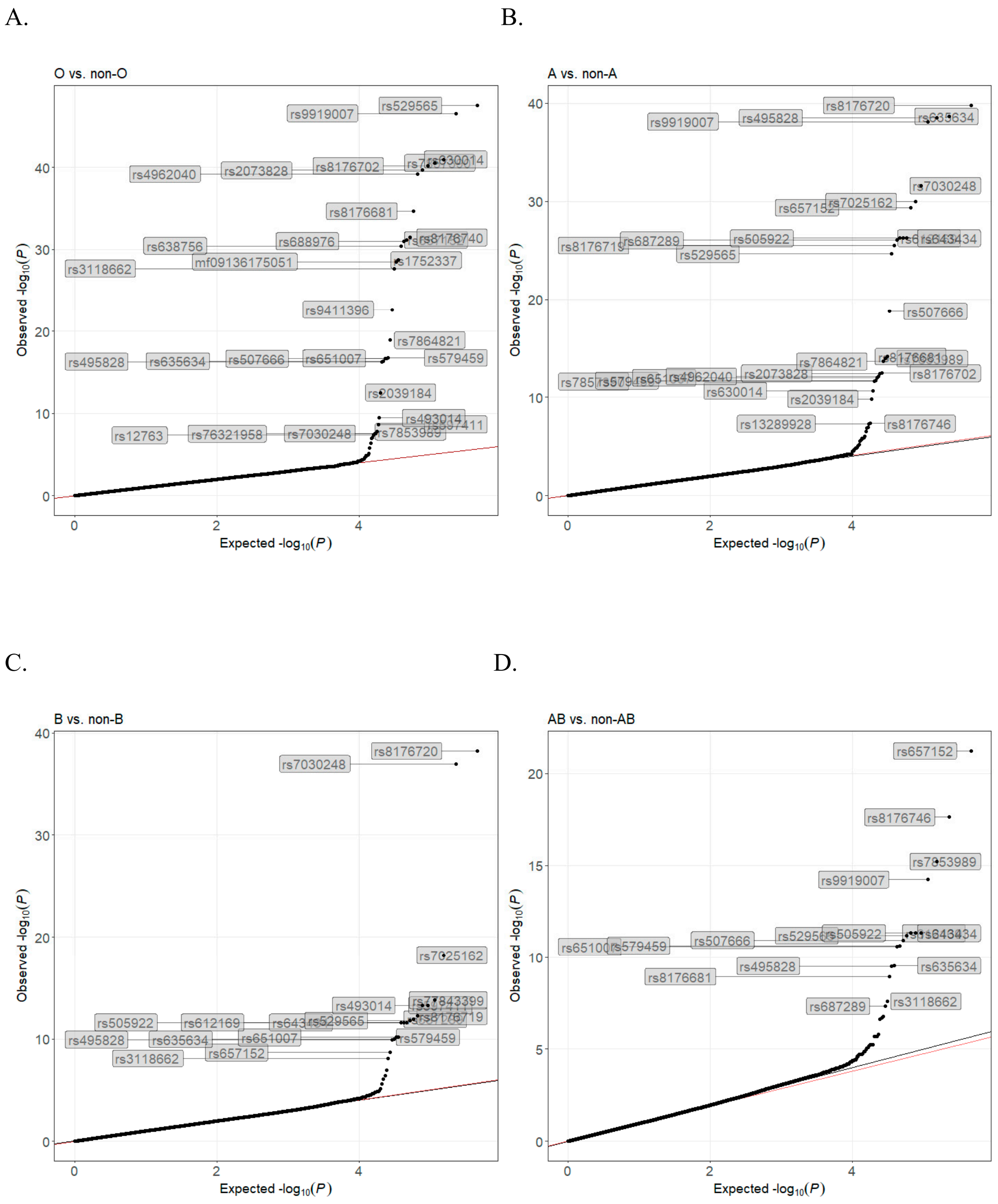
A.
O vs. non-O
B.
A vs. non-A
C.
B vs. non-B
D.
AB vs. non-AB
Observed -log10(P)
Expected -log10(P)
rs529565
rs9919007
rs4962040
rs2073828
rs8176702
rs630014
rs8176681
rs638756
rs688976
rs8176740
rs3118662
mf09136175051
rs1752337
rs9411396
rs7864821
rs495828
rs635634
rs507666
rs651007
rs579459
rs2039184
rs493014
rs12763
rs76321958
rs7030248
rs8176720
rs7025162
rs505922
rs687289
rs8176746
rs13289928
rs8176719
rs612169
rs657152
rs7853989
rs8176702
rs643434

Figure S4

A.

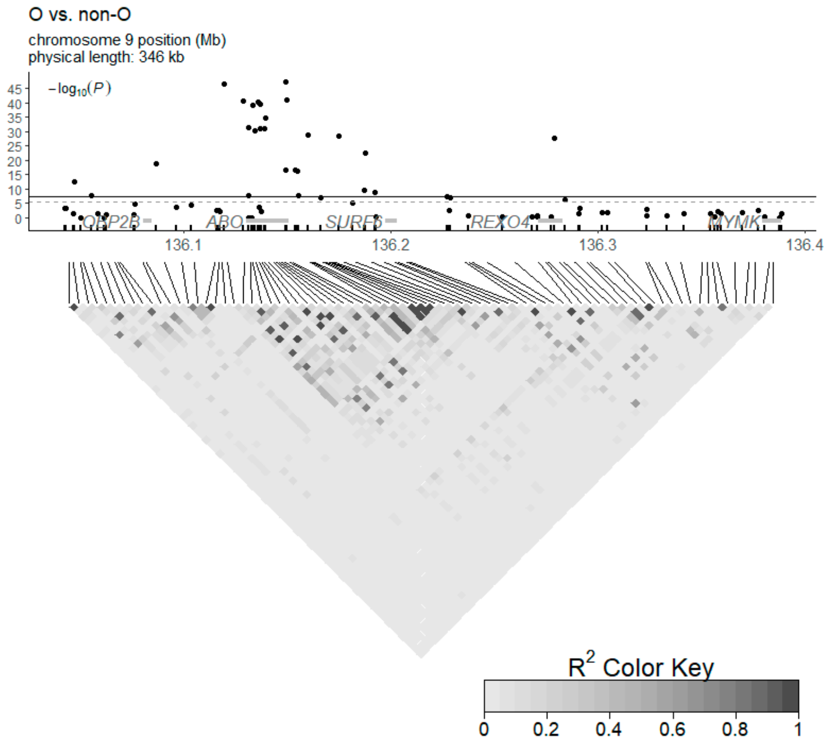
O vs. non-O
chromosome 9 position (Mb)
physical length: 346 kb
−log10(P)
45
40
35
30
25
20
15
10
5
0
ORP2B
ABO
SURF6
REXO4
MYMK
136.1
136.2
136.3
136.4
R2 Color Key
0
0.2
0.4
0.6
0.8
1

B.

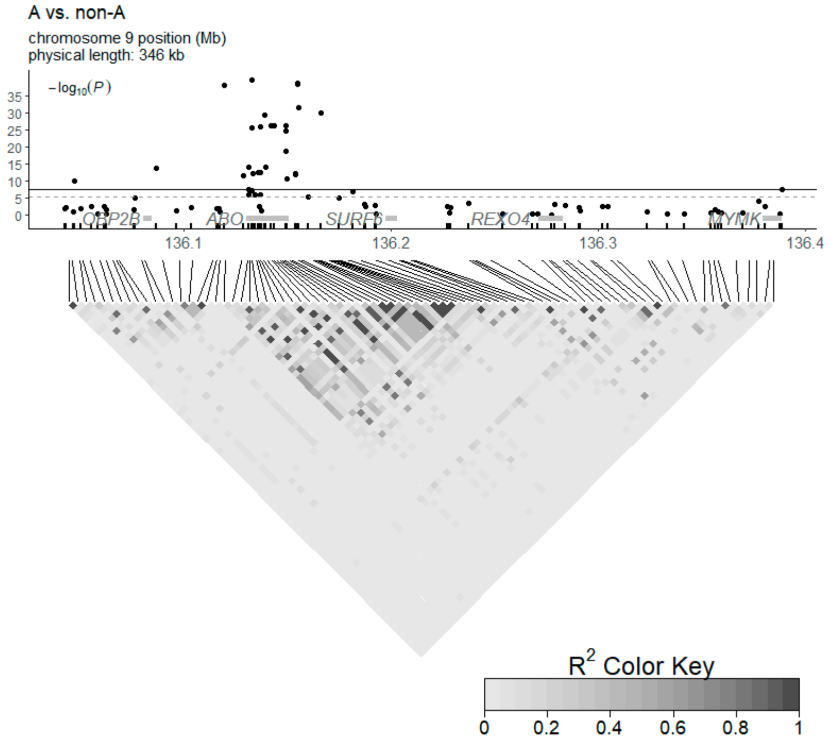
A vs. non-A
chromosome 9 position (Mb)
physical length: 346 kb
−log10(P)
35
30
25
20
15
10
5
0
ORP2B
ABO
SURF6
REXO4
MYMK
136.1
136.2
136.3
136.4
R2 Color Key
0
0.2
0.4
0.6
0.8
1

C.

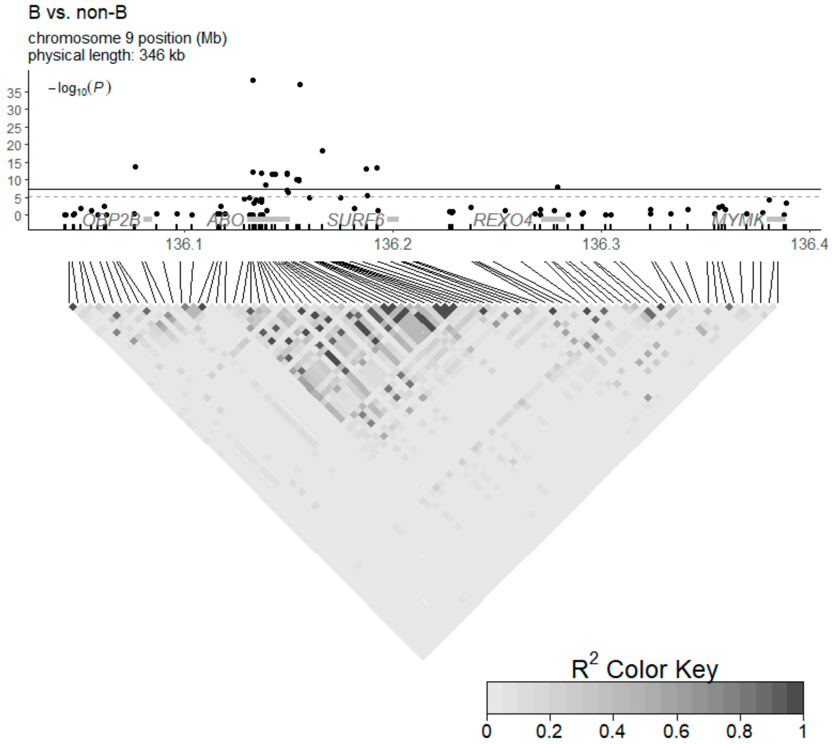
B vs. non-B
chromosome 9 position (Mb)
physical length: 346 kb
−log10(P)
35
30
25
20
15
10
5
0
OBP2B
ABO
SURF6
REXO4
MYMK
136.1
136.2
136.3
136.4
R2 Color Key
0
0.2
0.4
0.6
0.8
1

D.

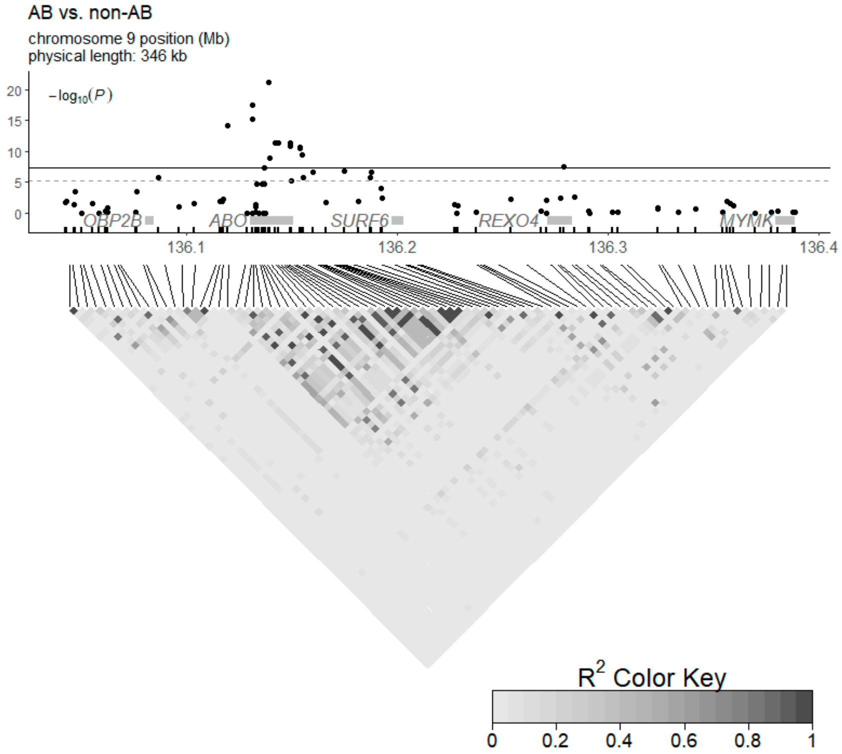
AB vs. non-AB
chromosome 9 position (Mb)
physical length: 346 kb
−log10(P)
20
15
10
5
0
OBP2B
ABO
SURF6
REXO4
MYMK
136.1
136.2
136.3
136.4
R2 Color Key
0
0.2
0.4
0.6
0.8
1

Figure S5

A.

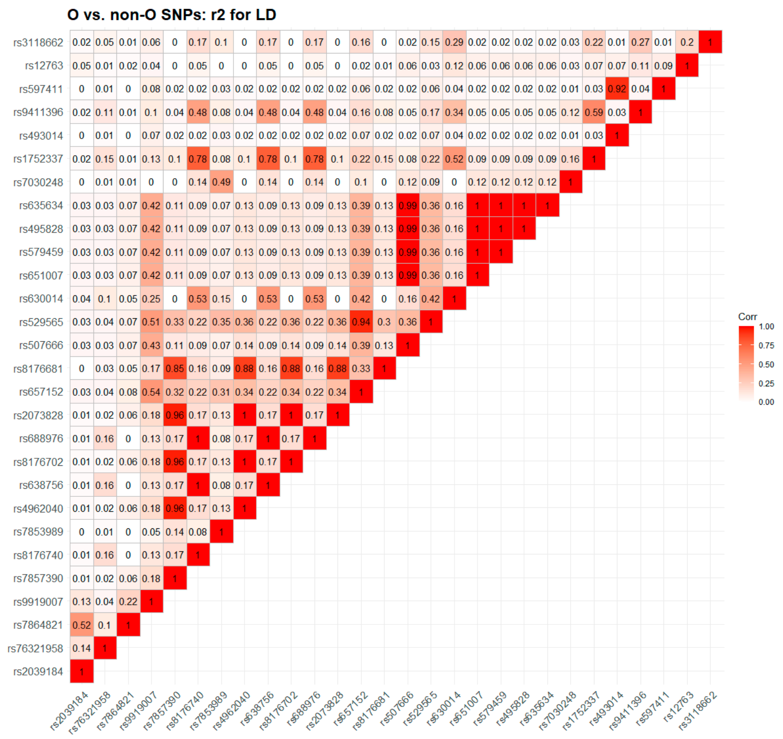

O vs. non-O SNPs: r2 for LD
rs3118662
rs12763
rs597411
rs9411396
rs493014
rs1752337
rs7030248
rs635634
rs495828
rs579459
rs651007
rs630014
rs529565
rs507666
rs8176681
rs657152
rs2073828
rs688976
rs8176702
rs638756
rs4962040
rs7853989
rs8176740
rs7857390
rs9919007
rs7864821
rs76321958
rs2039184
Corr
1.00
0.75
0.50
0.25
0.00

B.

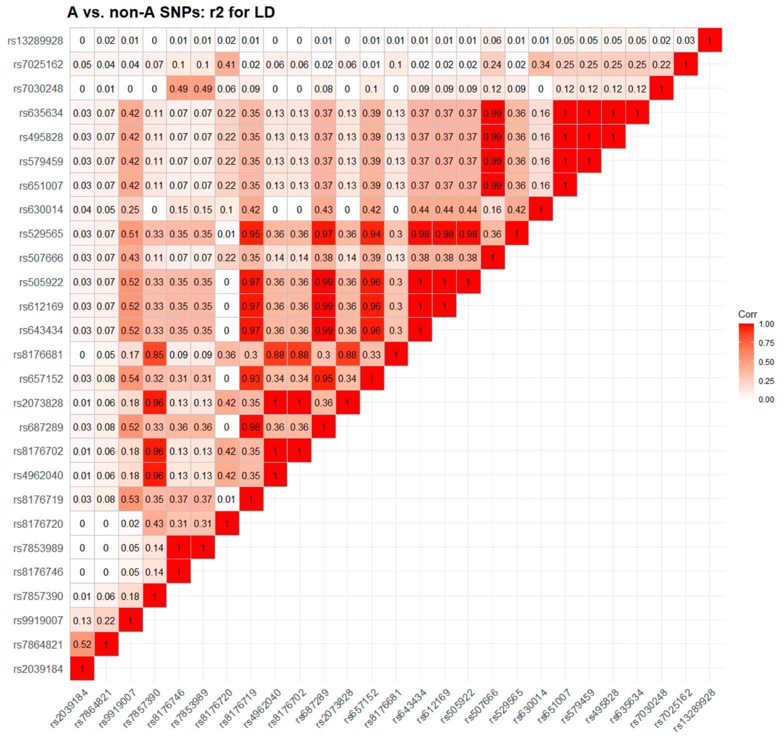

A vs. non-A SNPs: r2 for LD
rs13289928
rs7025162
rs7030248
rs635634
rs495828
rs579459
rs651007
rs630014
rs529565
rs507666
rs505922
rs612169
rs643434
rs8176681
rs657152
rs2073828
rs687289
rs8176702
rs4962040
rs8176719
rs8176720
rs7853989
rs8176746
rs7857390
rs9919007
rs7864821
rs2039184
Corr
1.00
0.75
0.50
0.25
0.00

C.

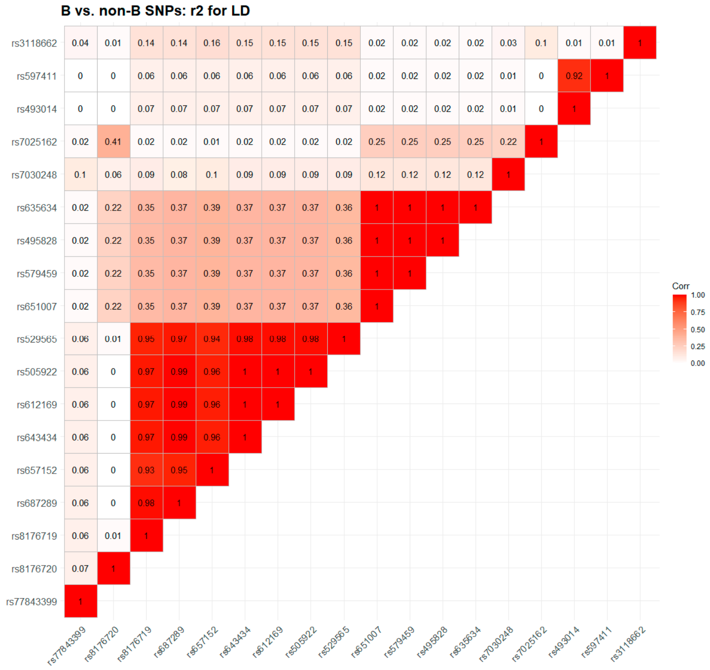
B vs. non-B SNPs: r2 for LD
rs3118662
rs597411
rs493014
rs7025162
rs7030248
rs635634
rs495828
rs579459
rs651007
rs529565
rs505922
rs612169
rs643434
rs657152
rs687289
rs8176719
rs8176720
rs77843399
rs77843399
rs8176720
rs8176719
rs687289
rs657152
rs643434
rs612169
rs505922
rs529565
rs651007
rs579459
rs495828
rs635634
rs7030248
rs7025162
rs493014
rs597411
rs3118662
Corr
1.00
0.75
0.50
0.25
0.00

D.

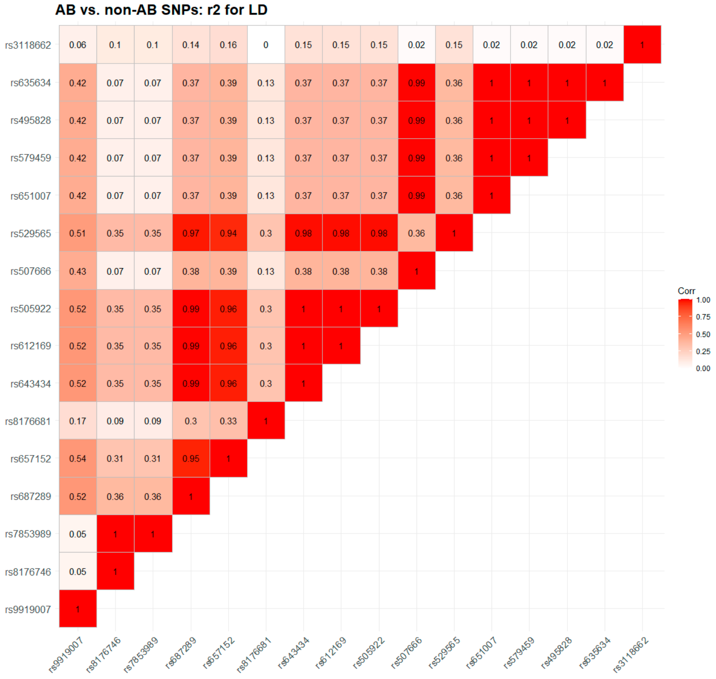
AB vs. non-AB SNPs: r2 for LD
rs3118662
rs635634
rs495828
rs579459
rs651007
rs529565
rs507666
rs505922
rs612169
rs643434
rs8176681
rs657152
rs687289
rs7853989
rs8176746
rs9919007
rs9919007
rs8176746
rs7853989
rs687289
rs657152
rs8176681
rs643434
rs612169
rs505922
rs507666
rs529565
rs651007
rs579459
rs495828
rs635634
rs3118662
Corr
1.00
0.75
0.50
0.25
0.00

Figure S6

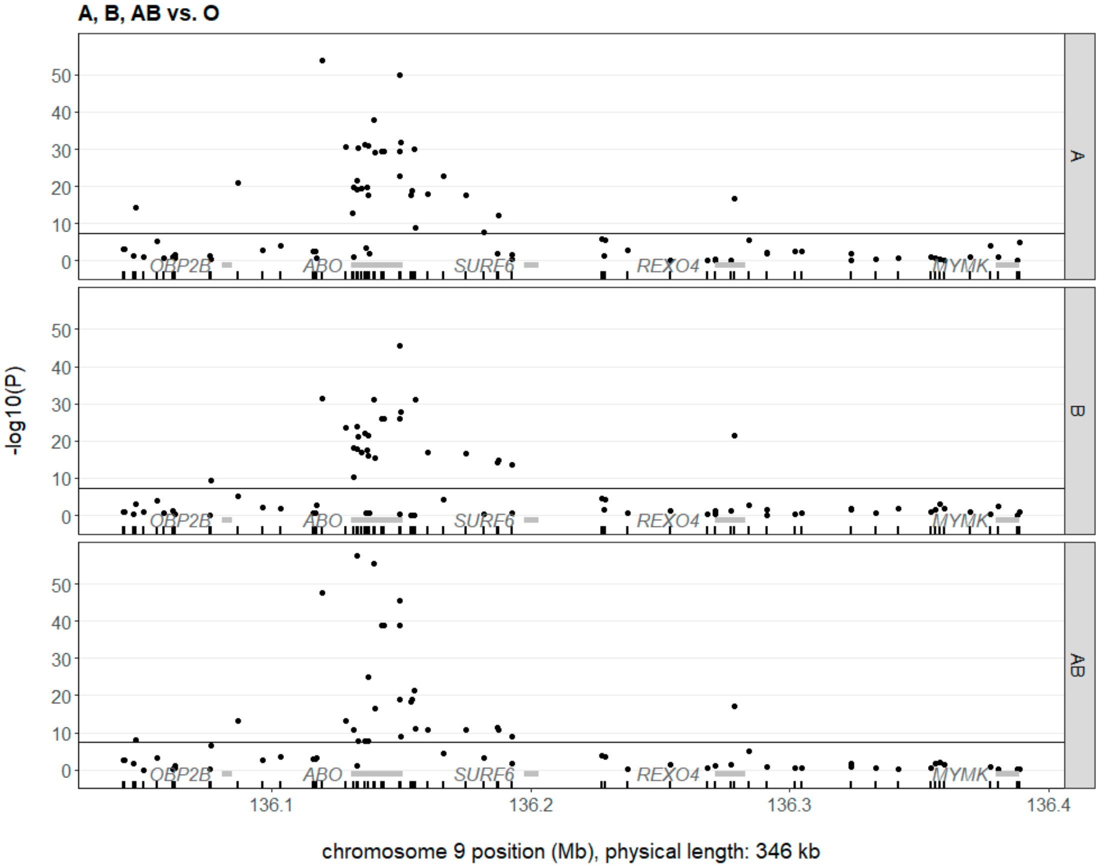
A, B, AB vs. O
-log10(P)
A
B
AB
OBP2B
ABO
SURF6
REXO4
MYMK
136.1
136.2
136.3
136.4
chromosome 9 position (Mb), physical length: 346 kb

Figure S7

A.

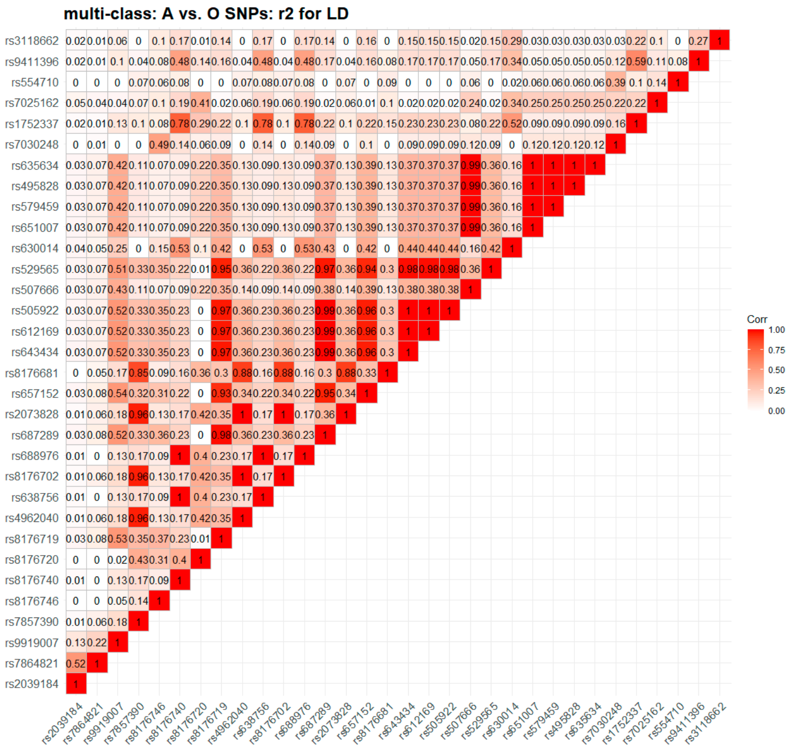
multi-class: A vs. O SNPs: r2 for LD
Corr
1.00
0.75
0.50
0.25
0.00

B.

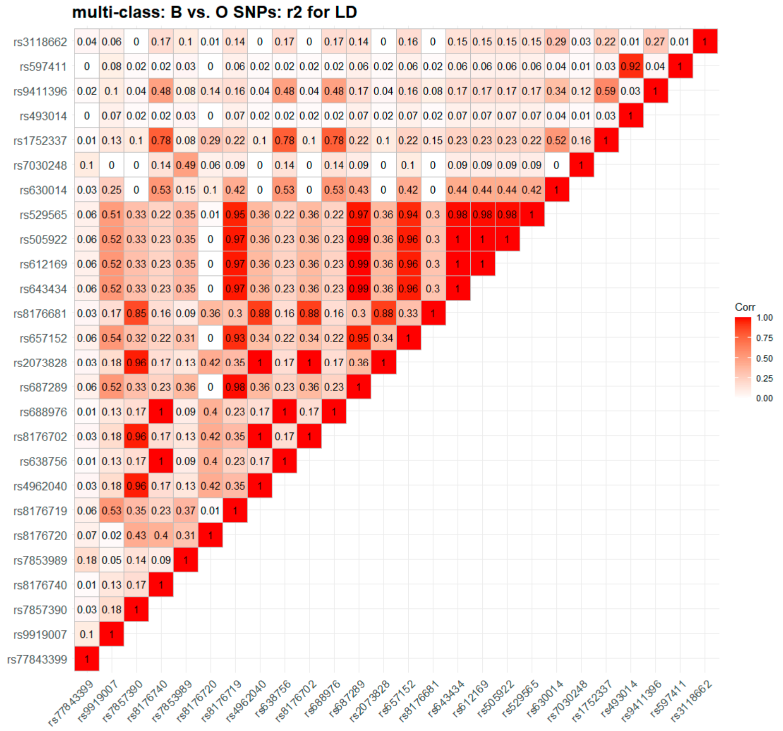
multi-class: B vs. O SNPs: r2 for LD
Corr
1.00
0.75
0.50
0.25
0.00

C.

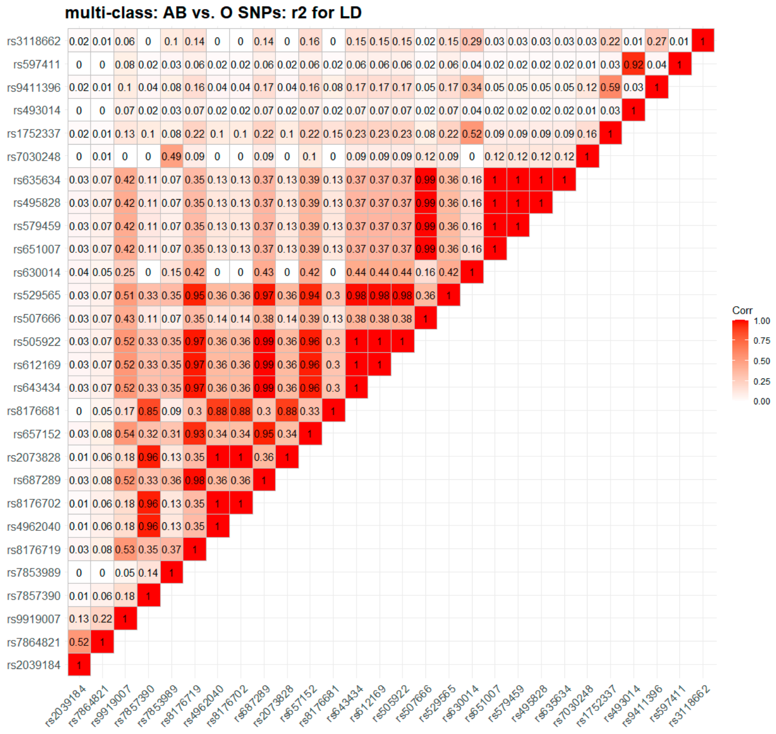
multi-class: AB vs. O SNPs: r2 for LD
rs3118662
rs597411
rs9411396
rs493014
rs1752337
rs7030248
rs635634
rs495828
rs579459
rs651007
rs630014
rs529565
rs507666
rs505922
rs612169
rs643434
rs8176681
rs657152
rs2073828
rs687289
rs8176702
rs4962040
rs8176719
rs7853989
rs7857390
rs9919007
rs7864821
rs2039184
Corr
1.00
0.75
0.50
0.25
0.00

Figure S8

A.

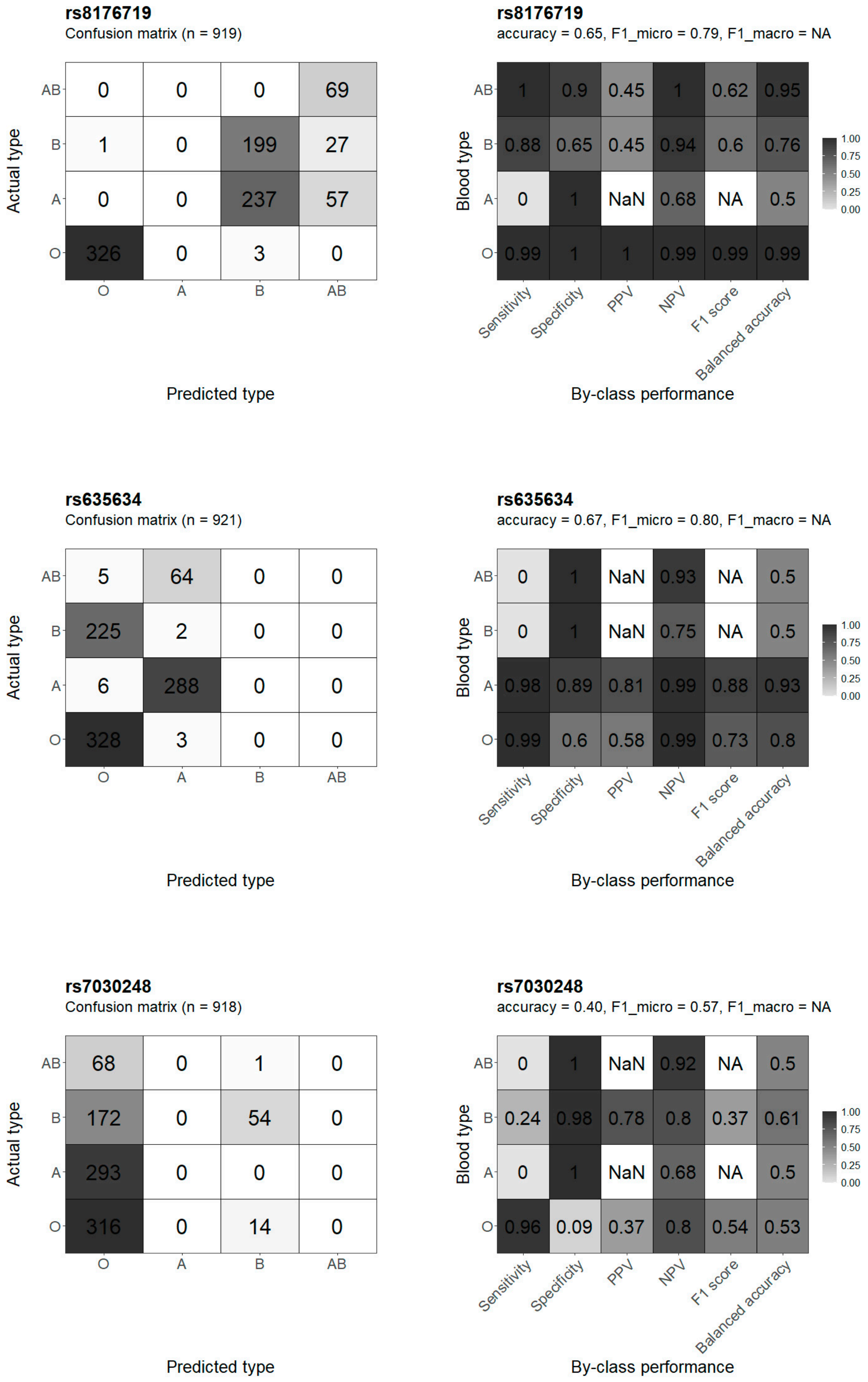
rs8176719
Confusion matrix (n = 919)
Actual type
AB 0 0 0 69
B 1 0 199 27
A 0 0 237 57
O 326 0 3 0
O A B AB
Predicted type
rs8176719
accuracy = 0.65, F1_micro = 0.79, F1_macro = NA
Blood type
AB 1 0.9 0.45 1 0.62 0.95
B 0.88 0.65 0.45 0.94 0.6 0.76
A 0 1 NaN 0.68 NA 0.5
O 0.99 1 1 0.99 0.99 0.99
Sensitivity Specificity PPV NPV F1 score Balanced accuracy
By-class performance
1.00 0.75 0.50 0.25 0.00
rs635634
Confusion matrix (n = 921)
Actual type
AB 5 64 0 0
B 225 2 0 0
A 6 288 0 0
O 328 3 0 0
O A B AB
Predicted type
rs635634
accuracy = 0.67, F1_micro = 0.80, F1_macro = NA
Blood type
AB 0 1 NaN 0.93 NA 0.5
B 0 1 NaN 0.75 NA 0.5
A 0.98 0.89 0.81 0.99 0.88 0.93
O 0.99 0.6 0.58 0.99 0.73 0.8
Sensitivity Specificity PPV NPV F1 score Balanced accuracy
By-class performance
1.00 0.75 0.50 0.25 0.00
rs7030248
Confusion matrix (n = 918)
Actual type
AB 68 0 1 0
B 172 0 54 0
A 293 0 0 0
O 316 0 14 0
O A B AB
Predicted type
rs7030248
accuracy = 0.40, F1_micro = 0.57, F1_macro = NA
Blood type
AB 0 1 NaN 0.92 NA 0.5
B 0.24 0.98 0.78 0.8 0.37 0.61
A 0 1 NaN 0.68 NA 0.5
O 0.96 0.09 0.37 0.8 0.54 0.53
Sensitivity Specificity PPV NPV F1 score Balanced accuracy
By-class performance
1.00 0.75 0.50 0.25 0.00

B.

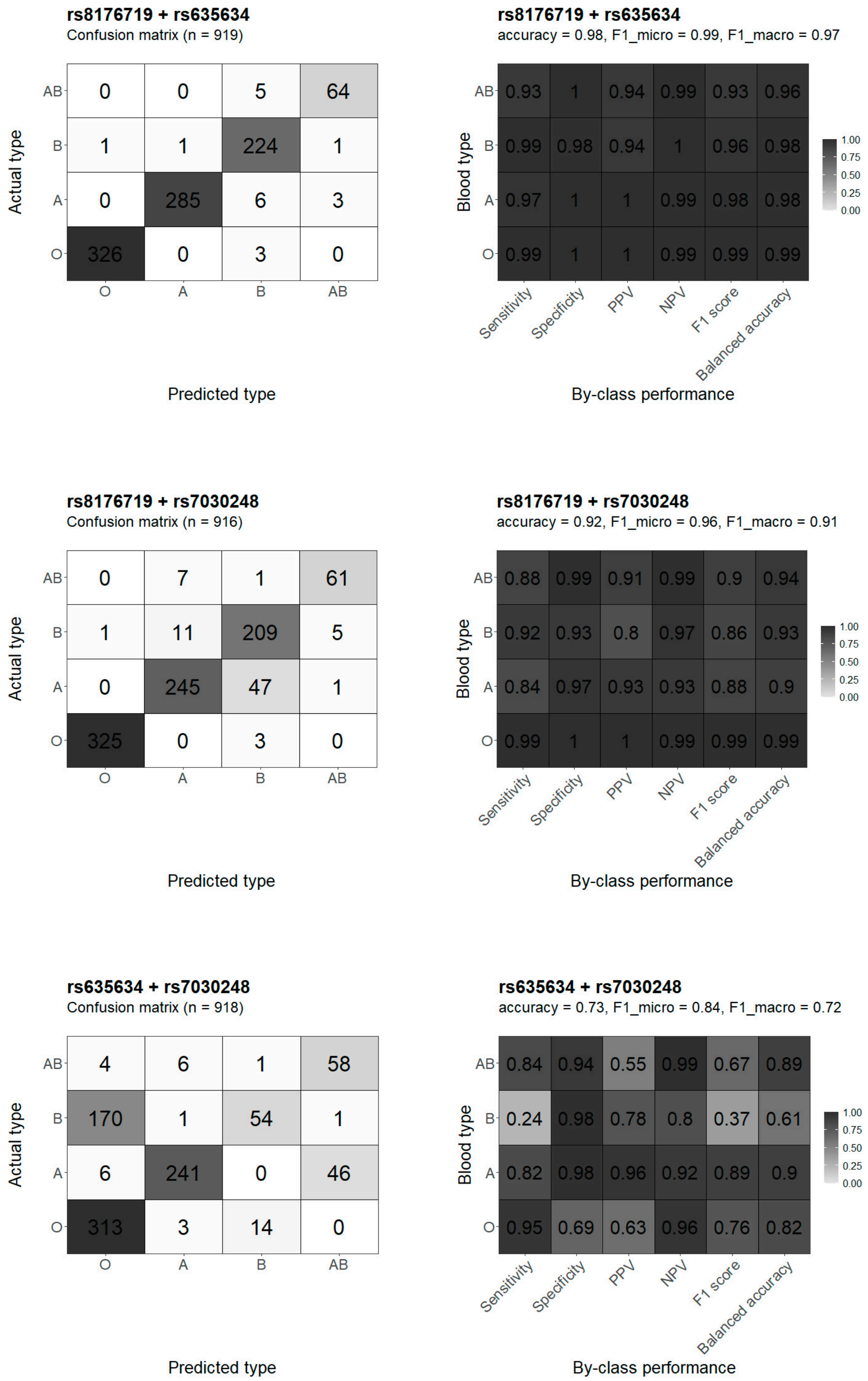

rs8176719 + rs635634
Confusion matrix (n = 919)
Actual type
Predicted type
rs8176719 + rs635634
accuracy = 0.98, F1_micro = 0.99, F1_macro = 0.97
Blood type
By-class performance
rs8176719 + rs7030248
Confusion matrix (n = 916)
Actual type
Predicted type
rs8176719 + rs7030248
accuracy = 0.92, F1_micro = 0.96, F1_macro = 0.91
Blood type
By-class performance
rs635634 + rs7030248
Confusion matrix (n = 918)
Actual type
Predicted type
rs635634 + rs7030248
accuracy = 0.73, F1_micro = 0.84, F1_macro = 0.72
Blood type
By-class performance
O
A
B
AB
Sensitivity
Specificity
PPV
NPV
F1 score
Balanced accuracy

C.

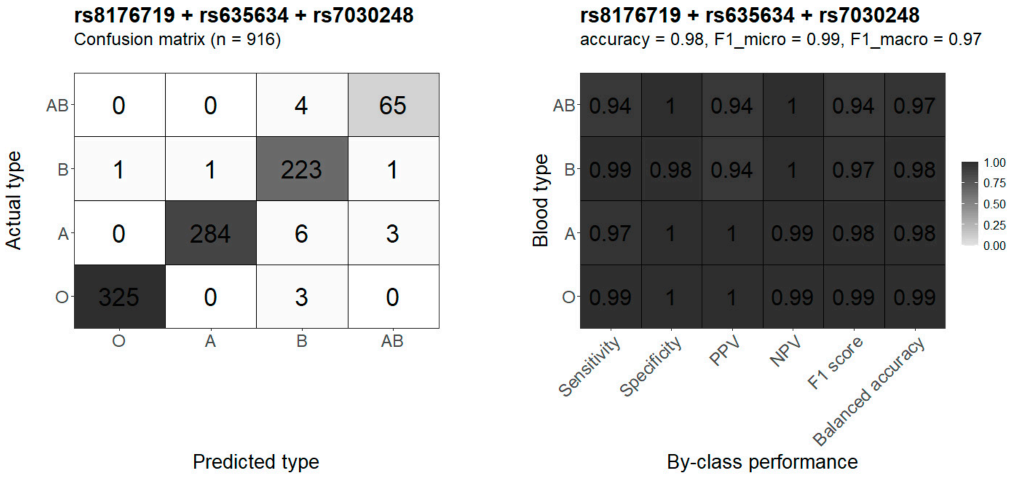

rs8176719 + rs635634 + rs7030248
Confusion matrix (n = 916)
Actual type
Predicted type
AB
B
A
O
O
A
B
AB
0
0
4
65
1
1
223
1
0
284
6
3
325
0
3
0
rs8176719 + rs635634 + rs7030248
accuracy = 0.98, F1_micro = 0.99, F1_macro = 0.97
Blood type
By-class performance
Sensitivity
Specificity
PPV
NPV
F1 score
Balanced accuracy
0.94
1
0.94
1
0.94
0.97
0.99
0.98
0.94
1
0.97
0.98
0.97
1
1
0.99
0.98
0.98
0.99
1
1
0.99
0.99
0.99
1.00
0.75
0.50
0.25
0.00

Figure S9

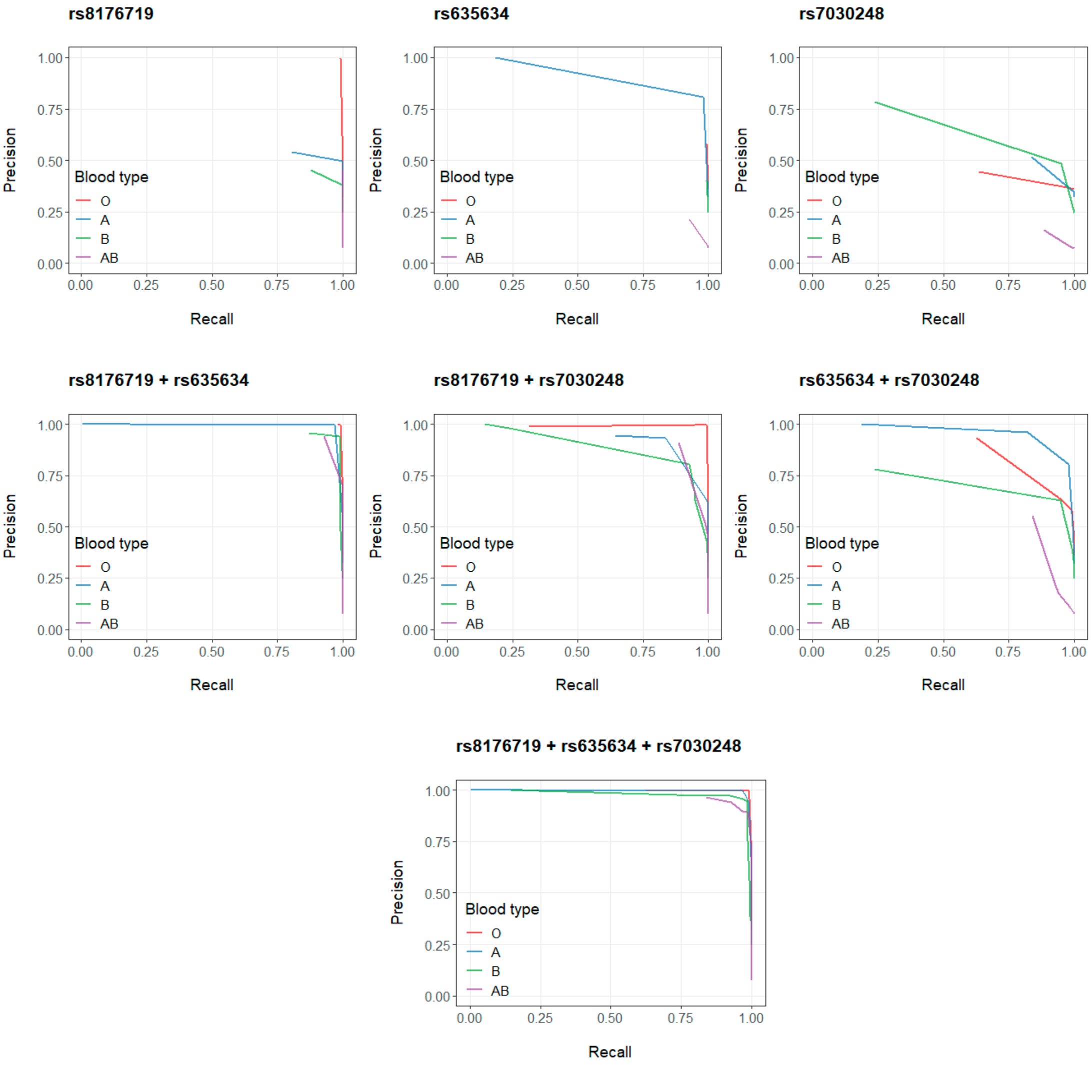
rs8176719
rs635634
rs7030248
rs8176719 + rs635634
rs8176719 + rs7030248
rs635634 + rs7030248
rs8176719 + rs635634 + rs7030248
Precision
Recall
1.00
0.75
0.50
0.25
0.00
0.00
0.25
0.50
0.75
1.00
Blood type
O
A
B
AB

Figure S10

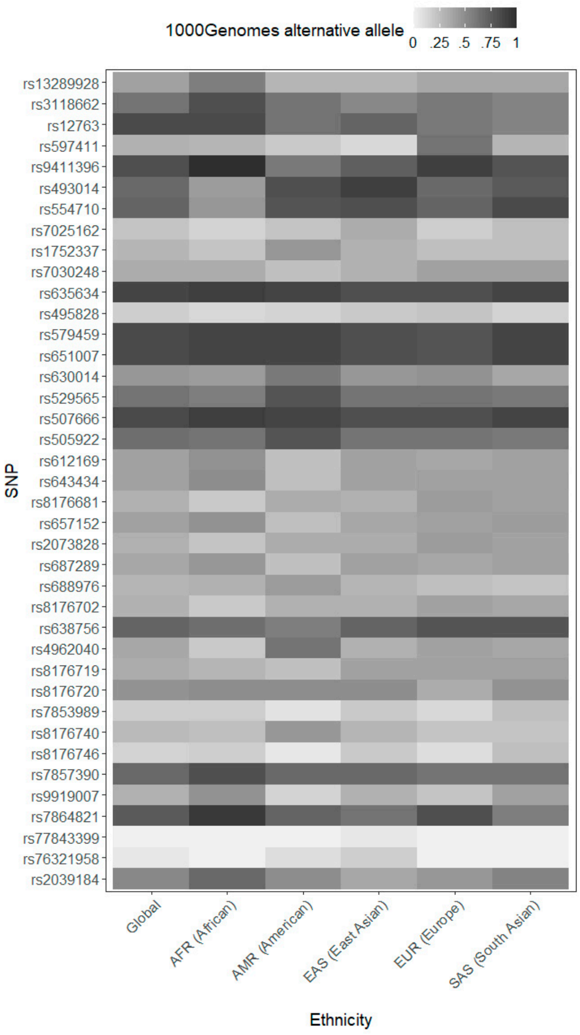
1000Genomes alternative allele
0
.25
.5
.75
1
SNP
rs13289928
rs3118662
rs12763
rs597411
rs9411396
rs493014
rs554710
rs7025162
rs1752337
rs7030248
rs635634
rs495828
rs579459
rs651007
rs630014
rs529565
rs507666
rs505922
rs612169
rs643434
rs8176681
rs657152
rs2073828
rs687289
rs688976
rs8176702
rs638756
rs4962040
rs8176719
rs8176720
rs7853989
rs8176740
rs8176746
rs7857390
rs9919007
rs7864821
rs77843399
rs76321958
rs2039184
Global
AFR (African)
AMR (American)
EAS (East Asian)
EUR (Europe)
SAS (South Asian)
Ethnicity

Supplementary figure legends

Figure S1. Schematic diagram for the study design of GWAS on ABO blood types in a Chinese population. QC: quality control; PCs: principal components.

Figure S2. Manhattan plots for the binary (2-level) outcomes: O *vs.* non-O (A), A *vs.* non-A (B), B *vs.* non-B (C) and AB *vs.* non-AB (D) in the cohort. Genome-wide hit SNPs (above the horizontal line) are annotated.

Figure S3. Quantile-quantile plots for the binary (2-level) outcomes: O *vs.* non-O (A), A *vs.* non-A (B), B *vs.* non-B (C) and AB *vs.* non-AB (D) in the cohort. Genome-wide hit SNPs are annotated. A line of $y = x$ (black) and a line of $y = \lambda_{median}x$ (red, the inflation factor λ_{median} are 1.008, 1.023, 1.013 and 0.952 respectively) are displayed.

Figure S4. Regional association plots for the binary (2-level) outcomes: O *vs.* non-O (A), A *vs.* non-A (B), B *vs.* non-B (C) and AB *vs.* non-AB (D) in the cohort. Genes are annotated as grey rectangles.

Figure S5. Linkage analysis (r^2) between ABO-associated hit SNPs for the binary (2-level) outcomes: O *vs.* non-O (A), A *vs.* non-A (B), B *vs.* non-B (C) and AB *vs.* non-AB (D) in the Chinese (CHB + CHS) from the 1000 Genomes (https://www.internationalgenome.org/).

Figure S6. Regional association plots for the quaternary (4-level, reference level of type O) outcomes of A *vs.* O (upper), B *vs.* O (middle) and AB *vs.* O (lower) in the cohort. Genes are annotated as grey rectangles.

Figure S7. Linkage analysis (r^2) between ABO-associated hit SNPs for the quaternary (4-level, reference level of type O) outcomes: A *vs.* O (A), B *vs.* O (B) and AB *vs.* O (C) in the Chinese (CHB + CHS) from the 1000 Genomes (https://www.internationalgenome.org/).

Figure S8. Confusion matrix (left) and classification performance parameters (right) for the prediction of ABO blood types based on *rs8176719*, *rs635634*, *rs7030248* (A), and their combinations (B and C). Accuracy, $F1_{micro}$, and $F1_{macro}$ evaluate the overall multi-class performances; the color shaded tiles evaluate the by-blood-type binary performances. $F1_{micro}$ and $F1_{macro}$ are the F1

scores designed for multi-class classification obtained by micro-averaging (weighted by class frequency) or macro-averaging (weighting all classes equally).

Figure S9. By-blood-type precision-recall plots for *rs8176719*, *rs635634*, *rs7030248*, and their combinations. Note: for precision-recall plots, scenario in which all individuals are predicted for the “negative” class results in 0/0 for precision and 0 for recall, thus unable to plot such data points (leading to the incomplete portion of plot); consequently, AUC (area under the curve) is not calculated for the precision-recall plots.

Figure S10. Allele frequency of common hit SNPs in the multinomial models from this study among the globe and five super-ancestries from the 1000 Genomes (https://www.internationalgenome.org/).

Supplementary tables

Table S1. Summary of the general and by-type baseline characteristics

	All	O	A	B	AB
n	1008	356	322	253	77
%	100	35.3	31.9	25.1	7.7
sex_{female}(%)	569(56.4)	194(54.5)	187(58.1)	142(56.1)	46(59.7)
age (years)					
- median	46	46	47	47	44
- IQR	39-52	39-52	39-53	39-52	36-52
- range	30-79	30-72	30-78	30-79	30-77
height (cm)					
- median	158	159	158	158	158
- IQR	153-164	154-164	153-164	154-164	152-166
- range	124-182	140-180	124-178	138-182	146-178
weight (kg)					
- median	59.8	60.0	59.8	59.2	58.6
- IQR	53.1-67.6	53.8-67.0	54.4-67.5	52.3-67.9	51.8-67.6
- range	35.5-111.2	37.2-111.2	39.5-101.4	35.5-106.1	43.7-90.0

Note: IQR - inter-quartile range.

Table S2. Distribution of ABO blood types across Asian ethnicities

Ethnicity	O (%)	A (%)	B (%)	AB (%)
Chinese (this study)	36	32	25	7
Chinese (Canton)	46	23	25	6
Chinese (Peking)	29	27	32	13
Japanese	30	38	22	10
Korean	28	32	31	10
Thai	37	22	33	8
Asian (in U.S.A - general)	40	28	27	5

Note: frequency of ABO blood types in ethnicities other than this study were retrieved from Wikipedia (https://en.wikipedia.org/wiki/Blood_type_distribution_by_country#cite_note-47, last accessed on November 2, 2020), which was referenced to "Racial and ethnic distribution of ABO blood types" curated by Bloodbook.com (http://www.bloodbook.com/world-abo.html, last accessed on November 2, 2020).

Table S10. Frequency summary of individuals with the defined set of SNPs (*n* = 916)

count	percentage (%)	rs8176719	rs635634	rs7030248
205	22.4	0	0	0
104	11.4	0	0	1
14	1.5	0	0	2
3	0.3	0	1	0
14	1.5	1	0	0
160	17.5	1	0	1
33	3.6	1	0	2
185	20.2	1	1	0
45	4.9	1	1	1
1	0.1	1	2	0
1	0.1	2	0	0
7	0.8	2	0	1
22	2.4	2	0	2
8	0.9	2	1	0
60	6.6	2	1	1
54	5.9	2	2	0

Note: 916 out of the 921 individuals subjected to GWAS analysis have all these three SNPs successfully genotyped.

Table S11. Summary of individuals with wrongly predicted ABO blood types (*n* = 19)

count	rs8176719	rs635634	rs7030248	blood-typed	predicted
4	1	0	0	A	B
3	2	0	1	AB	B
3	1	0	1	O	B
2	1	0	1	A	B
2	2	1	0	A	AB
1	0	0	1	B	O
1	2	1	1	B	AB
1	1	1	0	B	A
1	2	0	2	AB	B
1	2	1	1	A	AB

Table S3. Summary of GWAS on the binary trait of O *vs.* non-O blood types

SNP	Chr.(position)	genomic region	ra(%)	ma(%)	allele zygosity (%)	-log$_{10}$(P)	OR
O vs. non-O							
rs529565#	9(136149500)	ABO(intron)	C(59.4)	T(40.6)	CC(35.2),CT(48.4),TT(16.4)	47.5	0.0004
rs9919007#	9(136119527)	OBP2B*ABO	T(68.0)	C(32.0)	TT(46.8),TC(42.5),CC(10.7)	46.6	0.01
rs630014	9(136149722)	ABO(intron)	A(62.2)	G(37.8)	AA(37.9),AG(48.6),GG(13.5)	40.9	10.4
rs7857390	9(136128546)	ABO(3'-UTR)	A(66.4)	G(33.6)	AA(43.8),AG(45.0),GG(11.1)	40.5	7.5
rs8176702	9(136136146)	ABO(intron)	A(66.8)	G(33.2)	AA(44.7),AG(44.2),GG(11.1)	40.2	7.1
rs2073828	9(136137140)	ABO(intron)	A(66.4)	G(33.6)	AA(44.0),AG(45.0),GG(11.1)	39.6	7.0
rs4962040#	9(136133531)	ABO(intron)	G(66.9)	A(33.1)	GG(44.3),GA(45.2),AA(10.5)	39.2	7.0
rs8176681	9(136139754)	ABO(intron)	C(66.3)	T(33.7)	CC(43.8),CT(44.8),TT(11.4)	34.6	5.2
rs8176740#	9(136131472)	ABO(exon, F216I)	T(74.3)	A(25.7)	TT(54.6),TA(39.5),AA(5.9)	31.4	4.9
rs657152	9(136139265)	ABO(intron)	A(61.0)	C(39.0)	AA(37.1),AC(47.8),CC(15.1)	31.1	0.0003
rs688976#	9(136136770)	ABO(exon, V36F)	A(74.0)	C(25.9)	AA(54.3),AC(39.5),CC(6.2)	31.0	4.8
rs638756	9(136134472)	ABO(intron)	C(73.6)	A(26.4)	CC(53.5),CA(40.2),AA(6.3)	30.4	4.7
rs1752337#	9(136160228)	ABO*SURF6	G(72.3)	T(27.7)	GG(52.2),GT(40.2),TT(7.6)	28.7	4.2
mf09136175051	9(136175051)	ABO*SURF6	T(72.4)	C(27.6)	TT(52.2),TC(40.4),CC(7.4)	28.5	4.2
rs3118662#	9(136278870)	REXO4(intron)	G(51.6)	A(48.4)	GG(26.3),GA(50.7),AA(23)	27.6	3.8
rs9411396#	9(136187652)	ABO*SURF6	G(73.7)	T(26.3)	GG(53.8),GT(39.8),TT(6.4)	22.6	3.4
rs7864821#	9(136086916)	OBP2B(intron)	C(60.6)	T(39.4)	CC(36.8),CT(47.7),TT(15.5)	18.9	0.4
rs579459	9(136154168)	ABO*SURF6	C(77.6)	T(22.4)	CC(60.8),CT(33.7),TT(5.5)	16.8	0.007
rs651007#	9(136153875)	ABO*SURF6	T(77.4)	C(22.6)	TT(60.7),TC(33.5),CC(5.8)	16.7	0.007
rs507666	9(136149399)	ABO(intron)	A(77.8)	G(22.2)	AA(61.0),AG(33.6),GG(5.4)	16.6	0.007

rs495828	9(136154867)	ABO*SURF6	T(77.9)	G(22.1)	TT(61.5),TG(32.8),GG(5.7)	16.3	0.007
rs635634	9(136155000)	ABO*SURF6	T(77.9)	C(22.1)	TT(61.4),TC(32.9),CC(5.7)	16.3	0.007
rs2039184#	9(136047393)	RALGDS*OBP2B	G(59.2)	A(40.8)	GG(35.3),GA(48.0),AA(16.7)	12.5	0.4
rs493014#	9(136187163)	ABO*SURF6	G(90.7)	T(9.3)	GG(81.9),GT(17.5),TT(0.6)	9.4	0.2
rs597411	9(136192624)	ABO*SURF6	T(90.5)	G(9.5)	TT(81.7),TG(17.7),GG(0.6)	8.7	0.2
rs7853989#	9(136131592)	ABO(exon, R176G)	C(82.6)	G(17.4)	CC(66.4),CG(32.4),GG(1.2)	7.8	0.003
rs7030248#	9(136155359)	ABO*SURF6	A(71.9)	G(28.1)	AA(51.0),AG(41.8),GG(7.2)	7.6	0.5
rs76321958#	9(136055528)	RALGDS*OBP2B	C(84.6)	G(15.4)	CC(71.7),CG(25.8),GG(2.5)	7.5	2.1
rs12763#	9(136227260)	SURF2(exon,G213S)	A(67.7)	G(32.3)	AA(46.1),AG(43.2),GG(10.7)	7.3	0.5

Note: 1. coordinate position on chromosomes is based on GRCh37.

2. * in "genomic region" annotates intergenic region, e.g. OBP2B*ABO annotates an intergenic locus downstream of OBP2B and upstream of ABO.

3. ra - reference allele (major allele); ma - minor allele; OR, odds ratio.

4. the above notes also apply to Table S4 to S9.

5. # suffix in SNP annotates the subset of hit variants selected after LD screening ($r^2 < 0.8$) and to be evaluated for binary classification performances in Table 1.

For example, *rs529565* is the top ranked SNP for O *vs.* non-O and selected for binary classification evaluation (thus annotated with the suffix as *rs529565#*). With linkage analysis from Figure S5A, those hit variants with high linkage ($r^2 >= 0.8$) to *rs529565* would not be selected for binary classification evaluation (thus no # suffix: rs657152 with $r^2 = 0.94$). Iterating the above analysis for all hit SNPs leads to the selection of # suffix genetic variants to be evaluated in Table 1. This note also applies to Table S4 to S6.

Table S4. Summary of GWAS on the binary trait of A *vs.* non-A blood types

SNP	Chr.(position)	genomic region	ra(%)	ma(%)	allele zygosity (%)	$-log_{10}(P)$	OR
A vs. non-A							
rs8176720[#]	9(136132873)	ABO(exon, silent)	C(56.1)	T(43.9)	CC(30.4),CT(51.5),TT(18.1)	39.8	0.1
rs635634[#]	9(136155000)	ABO*SURF6	T(77.9)	C(22.1)	TT(61.5),TC(32.8),CC(5.7)	38.7	363.4
rs495828	9(136154867)	ABO*SURF6	T(77.9)	G(22.1)	TT(61.5),TG(32.8),GG(5.7)	38.5	358.6
rs9919007[#]	9(136119527)	OBP2B*ABO	T(68.0)	C(32.0)	TT(46.8),TC(42.6),CC(10.7)	38.1	6.4
rs7030248[#]	9(136155359)	ABO*SURF6	A(71.9)	G(28.1)	AA(51.0),AG(41.8),GG(7.2)	31.6	0.1
rs7025162[#]	9(136166346)	ABO*SURF6	T(60.2)	C(39.8)	TT(35.4),TC(49.8),CC(14.8)	30.0	4.6
rs657152	9(136139265)	ABO(intron)	A(61.0)	C(39.0)	AA(37.1),AC(47.8),CC(15.1)	29.4	4.2
rs505922	9(136149229)	ABO(intron)	C(59.3)	T(40.7)	CC(35),CT(48.7),TT(16.4)	26.2	3.6
rs612169	9(136143442)	ABO(intron)	G(59.2)	A(40.8)	GG(34.9),GA(48.8),AA(16.4)	26.2	3.6
rs643434	9(136142355)	ABO(intron)	A(59.2)	G(40.8)	AA(34.9),AG(48.8),GG(16.4)	26.2	3.6
rs687289	9(136137106)	ABO(intron)	A(59.5)	G(40.5)	AA(35.4),AG(48.2),GG(16.4)	26.0	3.6
rs8176719[#]	9(136132908)	ABO(exon, insert)	TC(59.4)	T(40.6)	II(35.0),ID(48.6),DD(16.4)	25.5	3.6
rs529565	9(136149500)	ABO(intron)	C(59.4)	T(40.6)	CC(35.2),CT(48.4),TT(16.4)	24.6	3.4
rs507666	9(136149399)	ABO(intron)	A(77.8)	G(22.2)	AA(61),AG(33.6),GG(5.4)	18.8	1302.9
rs8176681	9(136139754)	ABO(intron)	C(66.3)	T(33.7)	CC(43.8),CT(44.8),TT(11.4)	14.1	0.4
rs7853989[#]	9(136131592)	ABO(exon, R176G)	C(82.6)	G(17.4)	CC(66.4),CG(32.4),GG(1.2)	13.9	0.02
rs7864821[#]	9(136086916)	OBP2B(intron)	C(60.6)	T(39.4)	CC(36.8),CT(47.7),TT(15.5)	13.6	2.3
rs8176702	9(136136146)	ABO(intron)	A(66.8)	G(33.2)	AA(44.7),AG(44.2),GG(11.1)	12.5	0.4
rs2073828	9(136137140)	ABO(intron)	A(66.4)	G(33.6)	AA(44.0),AG(45.0),GG(11.0)	12.4	0.4
rs4962040	9(136133531)	ABO(intron)	G(66.9)	A(33.1)	GG(44.3),GA(45.2),AA(10.5)	12.1	0.4
rs651007	9(136153875)	ABO*SURF6	T(77.4)	C(22.6)	TT(60.7),TC(33.5),CC(5.8)	12.0	2330.0

rs579459	9(136154168)	ABO*SURF6	C(77.6)	T(22.4)	CC(60.8),CT(33.7),TT(5.5)	11.7	2471.6
rs7857390#	9(136128546)	ABO(3'-UTR)	A(66.4)	G(33.6)	AA(43.8),AG(45.1),GG(11.1)	11.6	0.4
rs630014#	9(136149722)	ABO(intron)	A(62.2)	G(37.8)	AA(37.9),AG(48.6),GG(13.5)	10.7	0.4
rs2039184#	9(136047393)	RALGDS*OBP2B	G(59.2)	A(40.8)	GG(35.3),GA(48.0),AA(16.8)	9.8	2.0
rs13289928#	9(136388702)	MYMK(intron)	G(72.0)	A(28.0)	GG(52.2),GA(39.6),AA(8.2)	7.3	1.8
rs8176746	9(136131322)	ABO(exon, L266M)	T(82.4)	G(17.6)	TT(67.3),TG(30.3),GG(2.4)	7.3	0.004

Table S5. Summary of GWAS on the binary trait of B *vs.* non-B blood types

SNP	Chr.(position)	genomic region	ra(%)	ma(%)	allele zygosity (%)	$-log_{10}(P)$	OR
B vs. non-B							
rs8176720#	9(136132873)	ABO(exon, silent)	C(56.1)	T(43.9)	CC(30.4),CT(51.5),TT(18.1)	38.3	8.5
rs7030248#	9(136155359)	ABO*SURF6	A(71.9)	G(28.1)	AA(51.0),AG(41.8),GG(7.2)	37.0	16.1
rs7025162#	9(136166346)	ABO*SURF6	T(60.2)	C(39.8)	TT(35.4),TC(49.8),CC(14.8)	18.2	0.3
rs77843399#	9(136076392)	RALGDS*OBP2B	T(95.8)	C(4.2)	TT(91.7),TC(8.2),CC(0.1)	13.8	7.3
rs493014#	9(136187163)	ABO*SURF6	G(90.7)	T(9.3)	GG(81.9),GT(17.5),TT(0.6)	13.3	3.8
rs597411	9(136192624)	ABO*SURF6	T(90.5)	G(9.5)	TT(81.7),TG(17.7),GG(0.6)	13.3	3.8
rs8176719#	9(136132908)	ABO(exon, insert)	TC(59.4)	T(40.6)	II(35.1),ID(48.6),DD(16.3)	12.2	2.3
rs687289	9(136137106)	ABO(intron)	A(59.5)	G(40.5)	AA(35.4),AG(48.2),GG(16.4)	11.9	2.3
rs529565	9(136149500)	ABO(intron)	C(59.4)	T(40.6)	CC(35.2),CT(48.4),TT(16.4)	11.8	2.3
rs505922	9(136149229)	ABO(intron)	C(59.3)	T(40.7)	CC(35.0),CT(48.6),TT(16.4)	11.6	2.2
rs612169	9(136143442)	ABO(intron)	G(59.2)	A(40.8)	GG(34.8),GA(48.8),AA(16.4)	11.6	2.2
rs643434	9(136142355)	ABO(intron)	A(59.2)	G(40.8)	AA(34.8),AG(48.8),GG(16.4)	11.6	2.2
rs579459	9(136154168)	ABO*SURF6	C(77.6)	T(22.4)	CC(60.8),CT(33.7),TT(5.5)	10.2	0.009
rs651007	9(136153875)	ABO*SURF6	T(77.4)	C(22.6)	TT(60.7),TC(33.5),CC(5.8)	10.2	0.009
rs635634#	9(136155000)	ABO*SURF6	T(77.9)	C(22.1)	TT(61.4),TC(32.9),CC(5.7)	10.0	0.01
rs495828	9(136154867)	ABO*SURF6	T(77.9)	G(22.1)	TT(61.5),TG(32.8),GG(5.7)	9.9	0.01
rs657152	9(136139265)	ABO(intron)	A(61.0)	C(39.0)	AA(37.1),AC(47.8),CC(15.1)	8.7	2.0
rs3118662#	9(136278870)	REXO4(intron)	G(51.6)	A(48.4)	GG(26.3),GA(50.7),AA(23.0)	8.1	0.5

Table S6. Summary of GWAS on the binary trait of AB *vs.* non-AB blood types

SNP	Chr.(position)	genomic region	ra(%)	ma(%)	allele zygosity (%)	$-\log_{10}(P)$	OR
AB vs. non-AB							
rs657152	9(136139265)	ABO(intron)	A(61.0)	C(39.0)	AA(37.1),AC(47.8),CC(15.1)	21.2	48.9
rs8176746#	9(136131322)	ABO(exon, L266M)	T(82.4)	G(17.6)	TT(67.3),TG(30.3),GG(2.4)	17.6	8.3
rs7853989	9(136131592)	ABO(exon, R176G)	C(82.6)	G(17.4)	CC(66.4),CG(32.4),GG(1.2)	15.2	13.0
rs9919007#	9(136119527)	OBP2B*ABO	T(68.0)	C(32.0)	TT(46.8),TC(42.6),CC(10.6)	14.2	4.8
rs505922	9(136149229)	ABO(intron)	C(59.3)	T(40.7)	CC(35.0),CT(48.7),TT(16.4)	11.3	199.9
rs612169	9(136143442)	ABO(intron)	G(59.2)	A(40.8)	GG(34.9),GA(48.8),AA(16.4)	11.3	199.9
rs643434	9(136142355)	ABO(intron)	A(59.2)	G(40.8)	AA(34.9),AG(48.8),GG(16.4)	11.3	199.9
rs529565	9(136149500)	ABO(intron)	C(59.4)	T(40.6)	CC(35.2),CT(48.4),TT(16.4)	11.2	208.1
rs507666#	9(136149399)	ABO(intron)	A(77.8)	G(22.2)	AA(61),AG(33.6),GG(5.4)	10.9	3.7
rs579459	9(136154168)	ABO*SURF6	C(77.6)	T(22.4)	CC(60.8),CT(33.7),TT(5.5)	10.6	3.5
rs651007	9(136153875)	ABO*SURF6	T(77.4)	C(22.6)	TT(60.7),TC(33.5),CC(5.8)	10.6	3.5
rs495828	9(136154867)	ABO*SURF6	T(77.9)	G(22.1)	TT(61.5),TG(32.8),GG(5.7)	9.5	3.2
rs635634	9(136155000)	ABO*SURF6	T(77.9)	C(22.1)	TT(61.4),TC(32.9),CC(5.7)	9.5	3.2
rs8176681#	9(136139754)	ABO(intron)	C(66.3)	T(33.7)	CC(43.8),CT(44.8),TT(11.4)	8.9	0.09
rs3118662#	9(136278870)	REXO4(intron)	G(51.6)	A(48.4)	GG(26.3),GA(50.7),AA(23.0)	7.6	0.3
rs687289#	9(136137106)	ABO(intron)	A(59.5)	G(40.5)	AA(35.4),AG(48.2),GG(16.4)	7.3	475.5

Table S7. Summary of GWAS on the quaternary trait of A *vs.* O blood types

SNP	Chr.(position)	genomic region	ra(%)	ma(%)	allele zygosity (%)	$-\log_{10}(P)$	OR
A vs. O							
rs9919007	9(136119527)	OBP2B*ABO	T(68.0)	C(32.0)	TT(46.8),TC(42.6),CC(10.7)	53.9	182.6
rs529565	9(136149500)	ABO(intron)	C(59.4)	T(40.6)	CC(35.2),CT(48.4),TT(16.4)	50.0	3.0×10^3
rs657152	9(136139265)	ABO(intron)	A(61.0)	C(39.0)	AA(37.1),AC(47.8),CC(15.1)	37.8	6.5×10^3
rs630014	9(136149722)	ABO(intron)	A(62.2)	G(37.8)	AA(37.9),AG(48.6),GG(13.5)	31.8	0.1
rs8176702	9(136136146)	ABO(intron)	A(66.8)	G(33.2)	AA(44.7),AG(44.2),GG(11.1)	31.1	0.1
rs2073828	9(136137140)	ABO(intron)	A(66.4)	G(33.6)	AA(44.0),AG(45.0),GG(11.0)	30.8	0.1
rs7857390	9(136128546)	ABO(3'-UTR)	A(66.4)	G(33.6)	AA(44.0),AG(45.0),GG(11.0)	30.7	0.1
rs4962040	9(136133531)	ABO(intron)	G(66.9)	A(33.1)	GG(44.3),GA(45.2),AA(10.5)	30.3	0.1
rs635634	9(136155000)	ABO*SURF6	T(77.9)	C(22.1)	TT(61.4),TC(32.9),CC(5.7)	30.1	1.1×10^4
rs495828	9(136154867)	ABO*SURF6	T(77.9)	G(22.1)	TT(61.4),TG(32.9),GG(5.7)	30.0	1.1×10^4
rs505922	9(136149229)	ABO(intron)	C(59.3)	T(40.7)	CC(35.0),CT(48.7),TT(16.4)	29.3	1.8×10^4
rs612169	9(136143442)	ABO(intron)	G(59.2)	A(40.8)	GG(34.9),GA(48.8),AA(16.4)	29.3	1.8×10^4
rs643434	9(136142355)	ABO(intron)	A(59.2)	G(40.8)	AA(34.9),AG(48.8),GG(16.4)	29.3	1.8×10^4
rs8176681	9(136139754)	ABO(intron)	C(66.3)	T(33.7)	CC(43.8),CT(44.8),TT(11.4)	29.2	0.2
rs507666	9(136149399)	ABO(intron)	A(77.8)	G(22.2)	AA(61.0),AG(33.6),GG(5.4)	22.9	4.8×10^4
rs7025162	9(136166346)	ABO*SURF6	T(60.2)	C(39.8)	TT(35.4),TC(49.8),CC(14.8)	22.7	4.3
rs8176720	9(136132873)	ABO(exon, silent)	C(56.1)	T(43.9)	CC(30.4),CT(51.5),TT(18.1)	21.5	0.2
rs7864821	9(136086916)	OBP2B(intron)	C(60.6)	T(39.4)	CC(36.8),CT(47.7),TT(15.5)	21.0	3.7
rs8176740	9(136131472)	ABO(exon, F216I)	T(74.3)	A(25.7)	TT(54.6),TA(39.5),AA(5.9)	19.9	0.2
rs688976	9(136136770)	ABO(exon, V36F)	A(74.1)	C(25.9)	AA(54.3),AC(39.5),CC(6.2)	19.6	0.2

rs638756	9(136134472)	ABO(intron)	C(73.6)	A(26.4)	CC(53.5),CA(40.2),AA(6.3)	19.4	0.2
rs8176719	9(136132908)	ABO(exon, insert)	TC(59.4)	T(40.6)	II(35.0),ID(48.6),DD(16.3)	19.1	2.4×10^{7}
rs579459	9(136154168)	ABO*SURF6	C(77.6)	T(22.4)	CC(60.8),CT(33.7),TT(5.5)	18.8	9.4×10^{4}
rs1752337	9(136160228)	ABO*SURF6	G(72.3)	T(27.7)	GG(52.2),GT(40.2),TT(7.6)	17.9	0.3
mf09136175051	9(136175051)	ABO*SURF6	T(72.4)	C(27.6)	TT(52.2),TC(40.4),CC(7.4)	17.7	0.3
rs651007	9(136153875)	ABO*SURF6	T(77.4)	C(22.6)	TT(60.7),TC(33.5),CC(5.8)	17.7	1.2×10^{5}
rs687289	9(136137106)	ABO(intron)	A(59.5)	G(40.5)	AA(35.4),AG(48.2),GG(16.4)	17.6	1.1×10^{5}
rs3118662	9(136278870)	REXO4(intron)	G(51.6)	A(48.4)	GG(26.3),GA(50.7),AA(23)	16.7	0.3
rs2039184	9(136047393)	RALGDS*OBP2B	G(59.2)	A(40.8)	GG(35.3),GA(48.0),AA(16.8)	14.4	2.7
rs8176746	9(136131322)	ABO(exon, L266M)	T(82.4)	G(17.6)	TT(67.3),TG(30.3),GG(2.4)	12.9	9.7×10^{5}
rs9411396	9(136187652)	ABO*SURF6	G(73.7)	T(26.3)	GG(53.8),GT(39.8),TT(6.4)	12.2	0.4
rs7030248	9(136155359)	ABO*SURF6	A(71.9)	G(28.1)	AA(51.0),AG(41.8),GG(7.2)	9.0	0.3
rs554710	9(136181848)	ABO*SURF6	C(83.3)	T(16.7)	CC(69.5),CT(27.6),TT(2.9)	7.6	0.4

Table S8. Summary of GWAS on the quaternary trait of B *vs.* O blood types

SNP	Chr.(position)	genomic region	ra(%)	ma(%)	allele zygosity (%)	$-\log_{10}(P)$	OR
B vs. O							
rs529565	9(136149500)	ABO(intron)	C(59.4)	T(40.6)	CC(35.2),CT(48.4),TT(16.4)	45.7	1.8×10^3
rs9919007	9(136119527)	OBP2B*ABO	T(68.0)	C(32.0)	TT(46.8),TC(42.6),CC(10.7)	31.5	41.1
rs657152	9(136139265)	ABO(intron)	A(61.0)	C(39.0)	AA(37.1),AC(47.8),CC(15.1)	31.1	2.2×10^3
rs7030248	9(136155359)	ABO*SURF6	A(71.9)	G(28.1)	AA(51.0),AG(41.8),GG(7.2)	31.0	17.3
rs630014	9(136149722)	ABO(intron)	A(62.2)	G(37.8)	AA(37.9),AG(48.6),GG(13.5)	27.9	0.1
rs505922	9(136149229)	ABO(intron)	C(59.3)	T(40.7)	CC(34.9),CT(48.8),TT(16.4)	26.1	8.9×10^3
rs612169	9(136143442)	ABO(intron)	G(59.2)	A(40.8)	GG(34.9),GA(48.8),AA(16.4)	26.1	8.9×10^3
rs643434	9(136142355)	ABO(intron)	A(59.2)	G(40.8)	AA(34.9),AG(48.8),GG(16.4)	26.1	8.9×10^3
rs8176720	9(136132873)	ABO(exon, silent)	C(56.1)	T(43.9)	CC(30.4),CT(51.5),TT(18.1)	23.7	6.2
rs7857390	9(136128546)	ABO(3'-UTR)	A(66.4)	G(33.6)	AA(43.8),AG(45),GG(11.1)	23.5	0.2
rs8176702	9(136136146)	ABO(intron)	A(66.8)	G(33.2)	AA(44.7),AG(44.2),GG(11.1)	22.2	0.2
rs2073828	9(136137140)	ABO(intron)	A(66.4)	G(33.6)	AA(44.0),AG(45.0),GG(11.0)	21.6	0.2
rs3118662	9(136278870)	REXO4(intron)	G(51.6)	A(48.4)	GG(26.3),GA(50.7),AA(23)	21.4	0.2
rs4962040	9(136133531)	ABO(intron)	G(66.9)	A(33.1)	GG(44.3),GA(45.2),AA(10.5)	21.2	0.2
rs8176740	9(136131472)	ABO(exon, F216I)	T(74.3)	A(25.7)	TT(54.6),TA(39.5),AA(5.9)	18.0	0.2
rs8176719	9(136132908)	ABO(exon, insert)	TC(59.4)	T(40.6)	II(35.1),ID(48.6),DD(16.3)	17.9	1.3×10^7
rs688976	9(136136770)	ABO(exon, V36F)	A(74.0)	C(25.9)	AA(54.3),AC(39.5),CC(6.2)	17.6	0.2
rs638756	9(136134472)	ABO(intron)	C(73.6)	A(26.4)	CC(53.5),CA(40.2),AA(6.3)	17.0	0.2
rs1752337	9(136160228)	ABO*SURF6	G(72.3)	T(27.7)	GG(52.2),GT(40.2),TT(7.6)	16.8	0.3
mf09136175051	9(136175051)	ABO*SURF6	T(72.4)	C(27.6)	TT(52.2),TC(40.4),CC(7.4)	16.7	0.3

rs687289	9(136137106)	ABO(intron)	A(59.5)	G(40.5)	AA(35.4),AG(48.2),GG(16.4)	15.9	6.0×10^{4}
rs8176681	9(136139754)	ABO(intron)	C(66.3)	T(33.7)	CC(43.8),CT(44.8),TT(11.4)	15.3	0.3
rs9411396	9(136187652)	ABO*SURF6	G(73.7)	T(26.3)	GG(53.8),GT(39.8),TT(6.4)	14.8	0.3
rs493014	9(136187163)	ABO*SURF6	G(90.7)	T(9.3)	GG(81.9),GT(17.5),TT(0.6)	14.2	7.8
rs597411	9(136192624)	ABO*SURF6	T(90.5)	G(9.5)	TT(81.7),TG(17.7),GG(0.6)	13.7	6.9
rs7853989	9(136131592)	ABO(exon, R176G)	C(82.6)	G(17.4)	CC(66.4),CG(32.4),GG(1.2)	10.3	3.9×10^{11}
rs77843399	9(136076392)	RALGDS*OBP2B	T(95.8)	C(4.2)	TT(91.7),TC(8.2),CC(0.1)	9.3	12.3

Table S9. Summary of GWAS on the quaternary trait of AB *vs.* O blood types

SNP	Chr.(position)	genomic region	ra(%)	ma(%)	allele zygosity (%)	$-\log_{10}(P)$	OR
AB vs. O							
rs8176719	9(136132908)	ABO(exon, insert)	TC(59.4)	T(40.6)	II(35.1),ID(48.6),DD(16.3)	57.7	1.4×10^{14}
rs657152	9(136139265)	ABO(intron)	A(61.0)	C(39.0)	AA(37.1),AC(47.8),CC(15.1)	55.4	2.2×10^{5}
rs9919007	9(136119527)	OBP2B*ABO	T(68.0)	C(32.0)	TT(46.8),TC(42.6),CC(10.7)	47.7	3.5×10^{2}
rs529565	9(136149500)	ABO(intron)	C(59.4)	T(40.6)	CC(35.2),CT(48.4),TT(16.4)	45.6	5.3×10^{5}
rs505922	9(136149229)	ABO(intron)	C(59.3)	T(40.7)	CC(35.0),CT(48.6),TT(16.4)	38.9	2.7×10^{6}
rs612169	9(136143442)	ABO(intron)	G(59.2)	A(40.8)	GG(34.9),GA(48.8),AA(16.4)	38.9	2.7×10^{6}
rs643434	9(136142355)	ABO(intron)	A(59.2)	G(40.8)	AA(34.9),AG(48.8),GG(16.4)	38.9	2.7×10^{6}
rs687289	9(136137106)	ABO(intron)	A(59.5)	G(40.5)	AA(35.4),AG(48.2),GG(16.4)	25.0	4.1×10^{7}
rs495828	9(136154867)	ABO*SURF6	T(77.9)	G(22.1)	TT(61.5),TG(32.8),GG(5.7)	21.2	1.4×10^{3}
rs635634	9(136155000)	ABO*SURF6	T(77.9)	C(22.1)	TT(61.4),TC(32.9),CC(5.7)	21.2	1.4×10^{3}
rs579459	9(136154168)	ABO*SURF6	C(77.6)	T(22.4)	CC(60.8),CT(33.7),TT(5.5)	18.9	3.7×10^{3}
rs507666	9(136149399)	ABO(intron)	A(77.8)	G(22.2)	AA(61.0),AG(33.6),GG(5.4)	18.8	3.2×10^{3}
rs651007	9(136153875)	ABO*SURF6	T(77.4)	C(22.6)	TT(60.7),TC(33.5),CC(5.8)	18.3	4.0×10^{3}
rs3118662	9(136278870)	REXO4(intron)	G(51.6)	A(48.4)	GG(26.3),GA(50.7),AA(23.0)	17.2	0.1
rs8176681	9(136139754)	ABO(intron)	C(66.3)	T(33.7)	CC(43.8),CT(44.8),TT(11.4)	16.5	0.03
rs7857390	9(136128546)	ABO(3'-UTR)	A(66.4)	G(33.6)	AA(43.8),AG(45),GG(11.1)	13.2	2.0×10^{-12}
rs7864821	9(136086916)	OBP2B(intron)	C(60.6)	T(39.4)	CC(36.8),CT(47.7),TT(15.5)	13.2	5.0
rs493014	9(136187163)	ABO*SURF6	G(90.7)	T(9.3)	GG(81.9),GT(17.5),TT(0.6)	11.4	9.6
rs7030248	9(136155359)	ABO*SURF6	A(71.9)	G(28.1)	AA(51.0),AG(41.8),GG(7.2)	11.0	6.3
rs9411396	9(136187652)	ABO*SURF6	G(73.7)	T(26.3)	GG(53.8),GT(39.8),TT(6.4)	10.8	0.06

mf09136175051	9(136175051)	ABO*SURF6	T(72.4)	C(27.6)	TT(52.2),TC(40.4),CC(7.4)	10.7	0.03
rs1752337	9(136160228)	ABO*SURF6	G(72.3)	T(27.7)	GG(52.2),GT(40.2),TT(7.6)	10.7	0.03
rs7853989	9(136131592)	ABO(exon, R176G)	C(82.6)	G(17.4)	CC(66.4),CG(32.4),GG(1.2)	10.7	1.2×10^{5}
rs597411	9(136192624)	ABO*SURF6	T(90.5)	G(9.5)	TT(81.7),TG(17.7),GG(0.6)	8.9	7.0
rs630014	9(136149722)	ABO(intron)	A(62.2)	G(37.8)	AA(37.9),AG(48.6),GG(13.5)	8.9	0.001
rs2039184	9(136047393)	RALGDS*OBP2B	G(59.2)	A(40.8)	GG(35.4),GA(48.0),AA(16.8)	8.2	3.2
rs4962040	9(136133531)	ABO(intron)	G(66.9)	A(33.1)	GG(44.3),GA(45.2),AA(10.5)	7.8	0.003
rs8176702	9(136136146)	ABO(intron)	A(66.8)	G(33.2)	AA(44.7),AG(44.2),GG(11.1)	7.8	0.003
rs2073828	9(136137140)	ABO(intron)	A(66.4)	G(33.6)	AA(44.0),AG(45.0),GG(11.0)	7.7	0.003
